# Micropropagation and Acclimatization of *Globba bicolor* Gagnep. with Phytochemical Profiling and Antioxidant Evaluation

**DOI:** 10.3390/biology15100743

**Published:** 2026-05-08

**Authors:** Surapon Saensouk, Phiphat Sonthongphithak, Thanchanok Dankasai, Theeraphan Chumroenphat, Sukanya Nonthalee, Nooduan Muangsan, Piyaporn Saensouk

**Affiliations:** 1 Walai Rukhavej Botanical Research Institute, Mahasarakham University, Maha Sarakham 44150, Thailand; surapon.s@msu.ac.th; 2 Diversity of Family Zingiberaceae and Vascular Plant for Its Applications Research Unit, Mahasarakham University, Maha Sarakham 44150, Thailand; phiphatmon@gmail.com; 3 Department of Biology, Faculty of Science, Mahasarakham University, Maha Sarakham 44150, Thailand; thanchanok210243@gmail.com; 4 Cosmetic Science and Spa Program, Faculty of Thai Traditional and Alternative Medicine, Ubon Ratchathani Rajabhat University, Ubon Ratchathani 34000, Thailand; theeraphan.c@ubru.ac.th; 5 Trang Horticultural Research Center, Horticultural Research Institute, Trang 92150, Thailand; sukanya_014@outlook.com; 6 School of Biology, Institute of Science, Suranaree University of Technology, Nakhon Ratchasima 30000, Thailand; nooduan@g.sut.ac.th

**Keywords:** bioactive compounds, flavonoid compounds, FTIR analysis, HPLC, plant regeneration, PLSR-model prediction, volatile oils, Zingiberaceae

## Abstract

*Globba bicolor* Gagnep., an ornamental ginger of cultural importance in Thailand’s “Tak Bat Dok Mai” festival, faces conservation challenges due to climate change and slow natural propagation. Limited understanding of its cultivation and chemical composition further constrains sustainable utilization. This study provides the first integrated investigation of micropropagation using rhizome-derived explants under various combinations of exogenous hormones, acclimatization strategies, and comparative phytochemical profiling between wild and in vitro-propagated plants. An optimized clonal regeneration system was established from plantlets, with Murashige and Skoog (MS) medium containing 2.0 mg/L 6-benzylaminopurine (BA) and 0.5 mg/L 1-naphthaleneacetic acid (NAA), yielding the highest multiplication (9.10 shoots/explant and 12.40 roots/explant) after eight weeks of cultivation. During acclimatization, sand substrate proved superior, facilitating a 90% survival rate and enhanced physiological vigor. Comparative analysis revealed that while wild plants possessed significantly higher total phenolic (TPC) and total flavonoid (TFC) contents and antioxidant activities (DPPH, ABTS, and FRAP) than their in vitro counterparts, both sources maintained a rich diversity of chemical constituents. HPLC analysis identified cinnamic acid, rutin, and quercetin as major metabolites, while GC–MS detected 90 volatile compounds, with β-caryophyllene and β-pinene as predominant constituents. Notably, rhizomes of wild plants exhibited particularly high-value detections. To provide a rapid and non-destructive approach for linking chemical composition with antioxidant activity, FTIR-based chemometric models were applied, demonstrating high predictive accuracy (*R*^2^–*cv* = 0.9712–0.9862). These results provide a scientific foundation for the conservation and sustainable commercial utilization of *G. bicolor* as a potential source of bioactive natural products.

## Simple Summary

*Globba bicolor* Gagnep. is a culturally important ornamental ginger in Thailand, yet its survival is increasingly under threat due to overharvesting driven by local use, climate change, and its naturally slow propagation rate. This study developed an efficient and practical micropropagation protocol that enables rapid plant multiplication and the successful establishment of healthy plants under ex vitro conditions. The secondary metabolites associated with potential health benefits were also investigated. Plants produced through this protocol showed good adaptability after transfer from in vitro to ex vitro environments. Phytochemical profiling further revealed abundant bioactive compounds together with strong antioxidant capacity. These findings demonstrate that micropropagation can effectively overcome propagation limitations while supporting the conservation of this culturally significant species and provide a foundation for developing *G. bicolor* as a valuable resource for pharmaceutical and horticultural applications.

## 1. Introduction

The ginger family (Zingiberaceae) is a large family of rhizomatous herbs, comprising more than 57 genera and approximately 2000 species distributed worldwide, with its greatest diversity occurring in Southeast Asia [1,2]. Several species of this family are highly valued for their diverse purposes, including use as food, spices, herbal medicines, and ornamental purposes [3,4,5]. Within this family, the genus *Globba* L., commonly known as dancing girl ginger, is particularly diverse in Thailand, encompassing more than 50 species [1,6], and has traditionally been utilized for medicinal, aromatic, and horticultural purposes [7,8,9].

**Figure 1:**
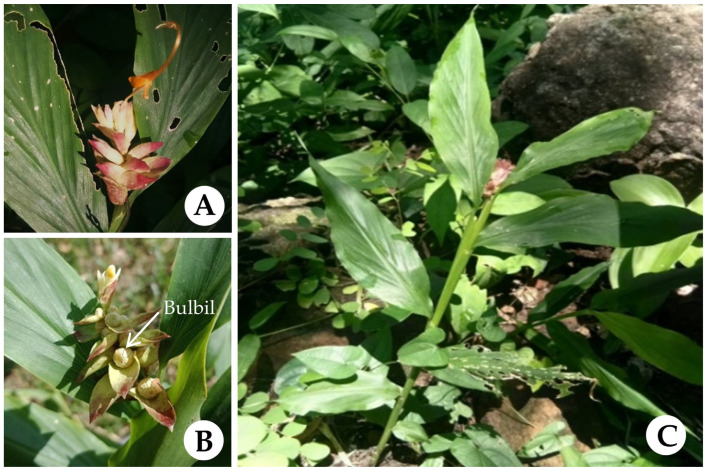
*Globba bicolor* Gagnep.; (**A**) inflorescence; (**B**) bulbils; (**C**) plant in habitat.

Within this genus, *Globba bicolor* Gagnep. (Figure 1) represents a notable species. The species was first discovered in 1901, with Cambodia designated as its type locality [10]. Subsequent records document its occurrence in Laos and Vietnam [6,11]. It is recognized as native to Thailand and was initially reported as a new record for the country in the northeastern region (Ubon Ratchathani Province), with the vernacular name Dok Khoa Punsa Song Sri [11]. It is now distributed across the northeastern, central, and southeastern regions of the country [6]. The inflorescences are culturally significant and are used in Buddhist rituals, particularly during the “Tak Bat Dok Mai” festival at Wat Phra Phutthabat in Saraburi Province [7]. In addition, members of the genus *Globba*, including this species, are valued as ornamental plants due to their distinctive inflorescences and colorful bracts [6,7,10,11,12]. This species is typically found in dry dipterocarp forests and limestone areas, where it grows in sandy loam soil. In Thailand, flowering is commonly observed during the rainy season [6,7]. It is a perennial herb that propagates mainly through rhizomes, with seasonal growth characterized by active development during the rainy period followed by a period of dormancy under unfavorable conditions [6,7]. Several species, including *Globba winitii*, *G. rosea*, *G. sherwoodiana*, and *G. schomburgkii* are widely cultivated and developed as ornamental plants for large-scale commercial production [13,14,15]. In addition, various parts of *Globba* species have been used in traditional medicine. For example, the inflorescences of *G. candida*, *G. obscura*, and *Globba* sp. (unidentified) have been employed to treat tuberculosis symptoms [12,16], while the rhizomes of *G. variabilis* Ridl. (syn. *G. malaccensis*) have been used to treat knee osteoarthritis [9]. The rhizomes of *G. conferta* are traditionally applied for skin diseases, whereas those of *G. hilaris* are used for gastrointestinal disorders [17].

*Globba* species are recognized for their rich phytochemical and volatile oil content, which serve as valuable resources for diverse applications. For instance, *G. marantina* contains pinocarvone, with *β*-caryophyllene being dominant in its rhizome [18]. The rhizome and aerial parts of *G. sessiliflora* also present abundant volatile compounds, including *β*-caryophyllene, myrcene, and selin-11-en-4α-ol [19]. Moreover, *G. candida*, *G. schomburgkii*, and *G. globulifera* are notable sources of volatile constituents with demonstrated antioxidant and other biological activities [20,21,22].

Despite their diverse applications, the commercial and pharmacological potential of *Globba* species is constrained by several factors, including limited growing and flowering periods, instability in vegetative traits, ecological limitations, conservation concerns, inconsistent yield performance, and absence of standardized scale-up cultivation protocols [23,24,25]. In addition, ongoing global warming poses increasing threats to plant biodiversity, particularly among species with restricted ecological tolerances and condensed reproductive cycles [26,27]. Therefore, developing strategies to overcome existing constraints and enhance propagation efficiency is crucial to increase plant availability and support multifaceted utilization across commercial, pharmacological, and conservation domains. In this context, plant tissue culture provides a robust and efficient approach to addressing these challenges. Through the establishment of a controlled environment for clonal propagation, it enables large-scale production, facilitates genetic preservation, and maintains stable vegetative traits. In contrast to conventional methods, tissue culture ensures morphological uniformity and consistent plantlet quality [28,29]. Furthermore, it has been demonstrated to be highly effective for the ex situ conservation and propagation of various *Globba* species [24,30,31,32,33,34,35,36], while simultaneously supporting the development of standardized large-scale cultivation systems [23,29].

To further evaluate the chemical quality and bioactive potential of the propagated plants, rapid analytical techniques are also required. Attenuated Total Reflectance Fourier Transform Infrared (ATR-FTIR) spectroscopy has emerged as a rapid and non-destructive analytical tool for characterizing plant metabolites [37]. By capturing unique vibrational fingerprints of functional groups, ATR-FTIR enables direct correlation between spectral features and antioxidant capacity. Recent studies highlight its potential for predicting phenolic content and radical-scavenging activities without the need for extensive chemical extractions [38,39]. Integrating ATR-FTIR into phytochemical research therefore provides a cost-effective and reproducible approach to evaluate antioxidant properties, supporting pharmaceutical applications of medicinal plants.

Although interest in *Globba* species has increased considerably, information on the in vitro propagation and phytochemical composition of *G. bicolor* remains limited, restricting its potential for broader applications. To address this gap, this study presents the first integrated approach combining micropropagation, acclimatization, comparative phytochemical profiling, and antioxidant evaluation between wild and in vitro-cultured plants. Furthermore, ATR-FTIR spectral data were applied to support phytochemical analysis and to predict antioxidant potential. This integrated approach provides a baseline framework for future research, conservation, and the sustainable utilization of *G. bicolor* as a potential ornamental plant and source of bioactive compounds.

## 2. Materials and Methods

### 2.1. Optimization of Micropropagation Protocol for Globba bicolor

#### 2.1.1. Explant Preparation and Culture Conditions

Rhizomes of mother plants, collected from Ubon Ratchathani Province, Thailand, were selected for initial propagation. The plant materials were identified and authenticated by Associate Professor Dr. Surapon Saensouk at the Walai Rukhavej Botanical Research Institute (WRBRI), Mahasarakham University, Thailand. Voucher specimens were deposited at the Vascular Plant Herbarium of Mahasarakham University (VMSU), Thailand. Prior to initial propagation, explants were decontaminated using a series of sodium hypochlorite solutions at concentrations of 20% and 10% (*v*/*v*) for 10 min, followed by rinsing with sterilized distilled water. The cleaned rhizomes were cultured on Murashige and Skoog (1962) (MS) medium [40] supplemented with 2 mg/L 6-benzylaminopurine (BA; Sigma–Aldrich, Budapest, Hungary) and 0.1 mg/L 1-naphthaleneacetic acid (NAA; Sd Fine–Chem Limited, Mumbai, India) to promote rapid propagation, following the method of Saensouk et al. [32]. Subsequently, the newly formed microshoots were subcultured after six weeks of growth onto MS medium without plant growth regulators (PGRs) to maintain stable explants. The culture conditions were maintained at 25 ± 2 °C with a 16 h photoperiod under a photosynthetic photon flux density of 27 μmol m^−2^ s^−1^, provided by fluorescent lamps (Philips TLD 36W/54-765 Cool Daylight, Bangkok, Thailand).

#### 2.1.2. Evaluation of the Combination of PGRs on *Globba bicolor* Propagation

**Figure 2:**
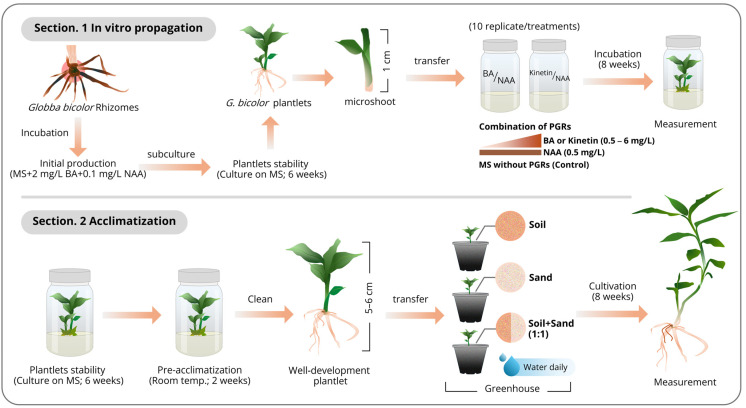
Illustration of the micropropagation and acclimatization workflow in *Globba bicolor*.

To evaluate the optimal medium composition for efficient propagation, stabilized plantlets in 1 cm length were transferred into MS medium supplemented with BA at concentrations ranging from 0.5 to 6 mg/L, along with 0.5 mg/L of NAA. Additionally, another type of cytokinin, kinetin (Sigma–Aldrich, Buchs, Switzerland), was tested for its propagation response using a combination of kinetin (0.5 to 6 mg/L) and 0.5 mg/L of NAA. MS medium without PGRs was used as the control for propagation. Each treatment was performed with ten replicates. Growth responses were recorded after 8 weeks of incubation, including the number of shoots per explant, shoot length (cm), number of roots per explant, and root length (cm). The experimental procedure is illustrated in Figure 2 (Section 1).

#### 2.1.3. Transplantation and Acclimatization

To establish the adaptation of *Globba bicolor* plantlets under ex vitro conditions, in vitro propagated plantlets obtained from the previous experiment (cultured on MS medium without PGR and pre-acclimated at room temperature for two weeks prior to transfer to potting substrate), exhibiting uniform morphology and well-developed roots, were carefully removed from the culture vessels. Any residual medium adhering to the roots was gently rinsed off with distilled water. The plantlets were transplanted into pots containing different well-dried substrates, including soil, sand, and a soil–sand mixture (1:1). Each pot contained a single plantlet (5–6 cm in length) and was covered with a transparent plastic dome to maintain humidity during the first week. After removing the plastic dome, the plantlets were irrigated daily with tap water and maintained under greenhouse conditions at Mahasarakham University, Thailand, for up to eight weeks. The experiment was conducted with ten replicates for each substrate type. Furthermore, the Soil–Plant Analysis Development (SPAD) method was used to evaluate the relative index of chlorophyll content in plantlet leaves cultivated in different substrates, with growth parameters also recorded, according to Wicharuck et al. [41] and Saensouk et al. [36]. The experimental procedure is illustrated in Section 2, Figure 2.

### 2.2. Determination of Phytochemical Profiling and Antioxidant Activity in Globba bicolor

#### 2.2.1. Plant Material Preparation and Extraction

Whole plants of *Globba bicolor* were collected from Ubon Ratchathani Province, Thailand (approximately 4-month-old plants at the flowering stage; July 2021). Micropropagated plantlets were obtained on MS medium supplemented with 2 mg/L BA and 0.1 mg/L NAA after 8 weeks of culture (early vegetative stage). Both samples’ sources were cleaned, separated into various parts, and freeze-dried for 48 h to obtain dried material. The dried samples were ground into a fine powder for extraction. The extraction process was adopted from Chumroenphat et al. [42]. Subsequently, 0.2 g of sample powder was incubated with 20 mL of 80% (*v*/*v*) ethanol under continuous shaking (150 rpm, 37 °C, 15 h) and then filtered through Whatman^®^ No. 1 filter paper. The filtrate was stored in a refrigerator for further experiments.

#### 2.2.2. Evaluation of Total Phenolic Content (TPC) and Total Flavonoid Content (TFC)

The Folin–Ciocalteu reagent (Sigma–Aldrich, Munich, Germany) was used to determine the total phenolic content in various plant parts of *Globba bicolor*. The procedure was adapted from Saensouk et al. [36]. In brief, 20 µL of plant extract was mixed with 100 µL of Folin–Ciocalteu reagent (20% *v*/*v*) in a 96-well plate and incubated at room temperature for 5 min (dark conditions). Subsequently, 75 µL of 10% (*w*/*v*) sodium carbonate (Na_2_CO_3_) solution was added to the mixture. After 2 h of incubation, absorbance was measured at 750 nm using a UV–Vis microplate reader (Variokan™ LUX, Thermo Fisher Scientific Inc., Marsiling, Singapore). To evaluate the total flavonoid content, an aluminum chloride colorimetric method was performed according to Saensouk et al. [36]. Briefly, 100 µL of deionized water was mixed with 25 µL of plant extract in a 96-well plate. Then, 10 µL of a 5% (*w*/*v*) sodium nitrite (NaNO_2_) solution was added to the mixture, followed by incubation at room temperature for 5 min under dark conditions. Next, 15 µL of aluminum chloride hexahydrate (AlCl_3_·6H_2_O, 10% *w*/*v*) was added and incubated in the dark for 6 min. After the reaction, 100 µL of 1 M sodium hydroxide (NaOH), diluted with deionized water (1:1), was pipetted into the mixture to complete the reaction. The absorbance of the mixture was recorded at 510 nm using a UV–Vis microplate reader. TPC and TFC were determined using calibration curves. Gallic acid (Merck KGaA, Beijing, China) served as the standard for TPC, with results expressed as milligrams of gallic acid equivalent per gram of dry weight (mg GAE/g DW), while rutin (Sigma–Aldrich, Beijing, China) was used as the standard for TFC, with results expressed as milligrams of rutin equivalent per gram of dry weight (mg RE/g DW).

#### 2.2.3. Evaluation of Antioxidant Activity

1. DPPH Radical Scavenging Assay

The antioxidant activity of plant extracts was assessed using the 2,2-diphenyl-1-picrylhydrazyl (DPPH) assay, following the method of Saensouk et al. [36]. For the analysis, 20 µL of plant extract was pipetted into a 96-well plate containing 180 µL of 0.15 M DPPH solution (Alfa Aesar, Thermo Fisher Scientific, Waltham, MA, USA). The mixture was homogenized and incubated in the dark for 30 min. After incubation, the absorbance of the mixture was measured at 517 nm using a UV–Vis microplate reader. The free radical scavenging activity was expressed as the percentage of radical inhibition.

2. ABTS Radical Scavenging Assay

The ABTS radical scavenging assay was performed according to Chumroenphat et al. [43] with slight modifications. A fresh ABTS stock solution (2,2′-azinobis(3-ethylbenzothiazoline-6-sulfonic acid; Sigma–Aldrich, Munich, Germany) was prepared at a concentration of 7.00 mmol/L and mixed with 2.45 mmol/L potassium persulfate at a 1:2 (*v*/*v*) ratio. The mixture was incubated in the dark at room temperature for 16 h to generate the ABTS∙^+^ radical cation. For the assay, 280 μL of the ABTS∙^+^ solution was added to each well of a 96-well microplate containing 20 μL of plant extract. After incubation for 30 min in the dark, the absorbance was measured at 734 nm using a UV–Vis microplate reader. The ABTS radical scavenging activity was calculated and reported as the percentage inhibition of the ABTS radical.

3. Ferric Reducing Antioxidant Power (FRAP) Assay

The FRAP assay was conducted as described by Saensouk et al. [44]. The FRAP solution (Fe^3+^–2,4,6-tripyridyl-s-triazine (TPTZ) complex) was freshly prepared and incubated at 37 °C for 15 min prior to use. A 5 µL aliquot of plant extract was added to 180 µL of FRAP solution in a 96-well plate and incubated at 37 °C for 16 min. Absorbance was measured at 593 nm employing a UV–Vis microplate reader. Antioxidant activity was expressed as milligrams of ferrous sulfate equivalent per gram of dry weight (mg FeSO_4_/g DW), calculated from a ferrous sulfate (FeSO_4_) standard curve.

#### 2.2.4. Functional Group Characterization of Phytochemicals by Fourier Transform Infrared Spectroscopy (FTIR)

1. FTIR Measurements

Fourier Transform Infrared Spectroscopy (FTIR) was employed to examine the vibrational spectra associated with phytochemical functional groups in *Globba bicolor*. For rapid evaluation of the spectra, the method followed Chumroenphat et al. [43]. In short, dried materials from different plant parts described in Section 2.2.1 were analyzed using a UATR accessory with a Diamond/KRS–5 crystal composite (PerkinElmer, Waltham, MA, USA). Infrared spectra were recorded from 32 scans at a resolution of 4 cm^−1^ over a wavenumber range of 400–4000 cm^−1^.

2. FTIR Spectral Pre-Processing, Characterization, and Statistical Analyses

All raw spectral data were recorded in triplicate for analysis. FTIR spectra were baseline-corrected using the multipoint baseline correction method and subsequently normalized to the range [0, 1] (min–max normalization) with OriginPro software version 2018 (OriginLab Corporation, Northampton, MA, USA). The identification of corresponding functional groups was based on previous studies and standard infrared spectroscopy references [45]. Furthermore, fingerprint region spectra (800–1800 cm^−1^ and 2800–3800 cm^−1^) of different samples were prepared for spectral characterization and analyzed using principal component analysis (PCA). Hierarchical cluster analysis (HCA) was subsequently performed with the average linkage algorithm and correlation distance to evaluate the similarity in spectral patterns across samples, both analyses conducted with PAST 5.0.1 software [46]. These analyses were carried out following Durank and Depciuch [47], with slight modifications.

3. Prediction of Antioxidant Activity

Based on the FTIR spectral features, two informative regions were selected for chemometric analysis: 3800–2800 cm^−1^ corresponding mainly to functional group vibrations, and 1800–800 cm^−1^ representing the fingerprint region. These regions contain major absorption bands associated with phenolics and other antioxidant-related compounds. PLS regression models were constructed using each region separately and their combination to evaluate their influence on antioxidant prediction.

FTIR spectra were preprocessed by baseline correction, smoothing, and standard normal variate (SNV) transformation to reduce noise and scattering effects. Two informative spectral regions (3800–2800 and 1800–800 cm^−1^) were selected based on characteristic absorption bands. PCA was first applied for exploratory analysis and outlier detection. Subsequently, partial least squares regression (PLSR) models with leave-group-out cross-validation (LGO; *n* = 3) were constructed for each antioxidant assay following Johnson et al. [38] and Câmara et al. [48] with modifications. Model performance was evaluated using the coefficient of determination (R^2^) and root mean square error (RMSE) for calibration (RMSEC) and cross-validation (RMSECV). The optimal number of latent variables was determined based on the minimum RMSECV. All analyses were performed using Minitab software version 22 (Minitab Inc., State College, PA, USA). Additionally, the PLS regression coefficients were used to interpret the effect of specific FTIR wavenumbers on antioxidant prediction, allowing identification of chemical bands associated with model performance.

#### 2.2.5. Quantitative Analysis of Phenolic Acids and Flavonoid Compounds Using High-Performance Liquid Chromatography (HPLC) in *Globba bicolor*

Plant Extraction: Dried samples (Section 2.2.1) were extracted following the method of Chumroenphat et al. [43]. Briefly, 0.3 g of dried sample was mixed with 20 mL of acidified methanol (0.1% *v*/*v* HCl) and incubated in a shaking incubator at 150 rpm and 37 °C for 15 h. The resulting extract was filtered and stored at 4 °C until further analysis.

HPLC Analysis: The ethanolic extract of *Globba bicolor* was analyzed using a Shimadzu LC–20A series system (Shimadzu Corp., Tokyo, Japan) equipped with an LC–20AC pump and an SPD-M20A diode array detector. Chromatographic separation was performed on a C–18 column (4.6 mm × 250 mm, 5 µm; LUNA^®^, Phenomenex Inc., Torrance, CA, USA) with an extended guard column. Gradient elution was carried out using 0.1% (*v*/*v*) acetic acid (line A) and acetonitrile (line B) as the mobile phase over 60 min. Shimadzu LC Solution software version 2.13 (Shimadzu, Tokyo, Japan) was used for data acquisition. The gradient program and diode array detector wavelength range followed Siriamornpun and Kaewseejan [49]. Retention times (RTs) and peak spectra were identified by comparison with reference standards and quantified using the external standard method.

#### 2.2.6. Analysis of Volatile Compounds Using GC-MS in *Globba bicolor*

Plant Extraction: Fresh materials were extracted according to Saensouk et al. [50] and Jakobina et al. [51] with modification. Each sample (0.2 g of fresh plant material) was transferred into vials and sealed with an aluminum cap (Supelco, Bellefonte, PA, USA). Headspace solid-phase microextraction (HS-SPME) was performed using a DVB/C-WR/PDMS fiber (divinylbenzene/carboxene/polydimethylsiloxane). Volatile compounds were extracted at 80 °C for 10 min, following a 10 min pre-incubation at the extraction temperature.

Operating Conditions of GC–MS: Aromatic compounds in the samples were analyzed using a GC–MS QP-2010 series system (Shimadzu, Tokyo, Japan) following the method of Saensouk et al. [51]. Separation was performed on a fused-silica capillary column (Rtx-5Ms; 5% diphenyl/95% dimethylpolysiloxane, 30 m length, 0.25 mm inner diameter, 0.25 µm film thickness; Restek, Bellefonte, PA, USA). Helium was employed as the carrier gas at a flow rate of 1.0 mL/min with a split ratio of 1:5, and the injector temperature was maintained at 250 °C. The oven program started at 40 °C, increased to 250 °C at 5 °C/min, and was held isothermally at 250 °C for 10 min. The transfer line was maintained at 250 °C. Ionization was conducted in electron impact mode at 70 eV, and spectra were recorded over the mass range of 50–550 amu. Data acquisition and peak integration were performed using GC–MS Solution software version 2.53 (Shimadzu, Tokyo, Japan), with subsequent processing in Microsoft Excel 365 (Microsoft, Washington, DC, USA). Compound identification was achieved by comparison with mass spectral data from the NIST 11 libraries, the NIST Chemistry Webbook [52], and Adam’s reference book [53].

### 2.3. Statistical Analysis

In Vitro Propagation and Acclimatization: All treatments were arranged in a completely randomized design (CRD) to evaluate optimized plant production. Data were expressed as mean ± standard error (SE), based on ten replicates per treatment. Differences among groups were analyzed using one-way ANOVA (*p* < 0.05), and mean separation was determined with Duncan’s multiple range test (DMRT). The Shapiro–Wilk test was applied to assess normality, and homogeneity of variances was evaluated using Levene’s test. All statistical analyses were performed with SPSS software version 29 (IBM Corp., New York, NY, USA).

Phytochemical Profiling and Antioxidant Capacity Evaluation: Each treatment was replicated three times, and values are presented as mean ± standard error (SE). Prior to statistical analysis, the dataset was tested for normality and variance homogeneity. Differences among treatments were analyzed using one-way ANOVA, and DMRT was employed for post hoc comparisons at a significance level of *p* < 0.05. Associations between variables were examined using the Pearson correlation coefficient (*r*), which indicates both the strength and direction of relationships. Principal component analysis (PCA) was conducted to evaluate the relationships between total phenolic, total flavonoid composition and their antioxidant capacity. All statistical analyses were performed using the statistical software (SPSS software, version 29) specified in each respective section.

FTIR Spectra Analysis: Each measurement was performed in triplicate, and all spectra were subjected to baseline correction and normalization using OriginPro 2018 software.

## 3. Results

### 3.1. Micropropagation of Globba bicolor

#### 3.1.1. Effect of Combination of BA or Kinetin with NAA on Shoots and Roots Regeneration

**Figure 3:**
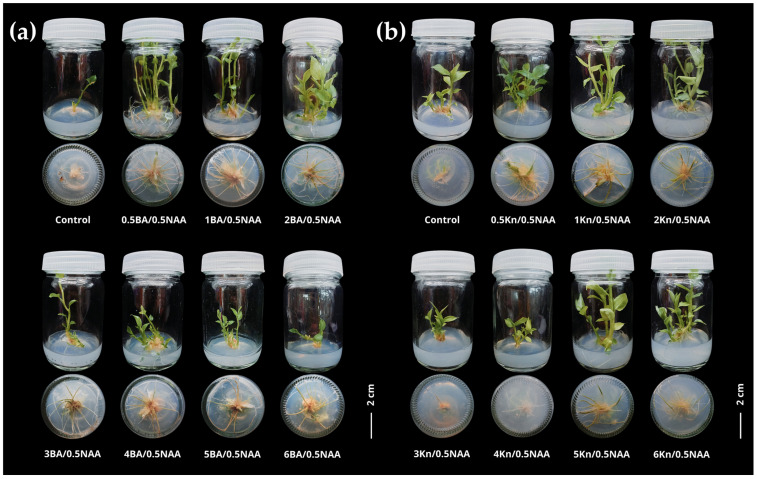
Shoots and roots regeneration of *Globba bicolor* cultured on media containing PGRS at different concentrations after 8 weeks of culture; (**a**) BA + NAA; (**b**) kinetin + NAA. scale bar = 2 cm.

Explants initially established in PGR-free medium were subsequently treated with different PGR combinations at varying concentrations for 8 weeks. Results showed that explants cultured without PGRs produced only 3.43–3.50 shoots/explant and 0.2–3.85 roots/explant, with short shoot (2.65–2.69 cm) and root lengths (0.38–1.42 cm) (Table 1 and Table 2 and Figure 3). In contrast, BA combined with NAA significantly promoted shoot and root induction. The treatment with 2.0 mg/L BA supplementation with 0.5 mg/L NAA gave the highest shoot induction (9.10 ± 1.30 shoots/explant) and longest roots (7.69 ± 0.71 cm) (Table 1 and Figure 3a), whereas 1.0 mg/L BA combined with 0.5 mg/L NAA yielded the highest number of roots (17.70 ± 1.56 roots/explant) (Table 1). At BA concentrations ≥ 3.0 mg/L, both shoot and root formation declined markedly, suggesting that excessive BA inhibited morphogenesis. Overall, moderate BA levels (1.0–2.0 mg/L) combined with 0.5 mg/L NAA were most effective for shoot and root development.

Similar to BA, kinetin in combination with NAA also promoted shoot and root induction, although the optimal concentrations differed slightly. Kinetin together with NAA also enhanced shoot and root development, yielding more shoots and roots compared with the control. The combination of 0.5 mg/L kinetin with 0.5 mg/L NAA resulted in the highest shoot production (6.40 ± 1.23 shoots/explant) and increased root formation (10.90 ± 2.50 roots/explant) (Table 2 and Figure 3b). Shoot elongation was maximized at 1.0 mg/L kinetin added to 0.5 mg/L NAA, which also produced the longest roots (6.12 ± 1.01 cm) and the highest number of roots produced (11.10 ± 2.80 roots/explant) (Table 2 and Figure 3b). At kinetin concentrations above 3.0 mg/L, both shoot and root responses decreased significantly, indicating an inhibitory effect on development.

Taken together, moderate cytokinin concentrations (1.0–2.0 mg/L), either BA or kinetin, combined with 0.5 mg/L NAA, provided optimal conditions for shoot and root induction in *Globba bicolor*, whereas higher cytokinin levels suppressed shoot and root proliferation.

#### 3.1.2. Acclimatization of *Globba bicolor* Plantlets

**Figure 4:**
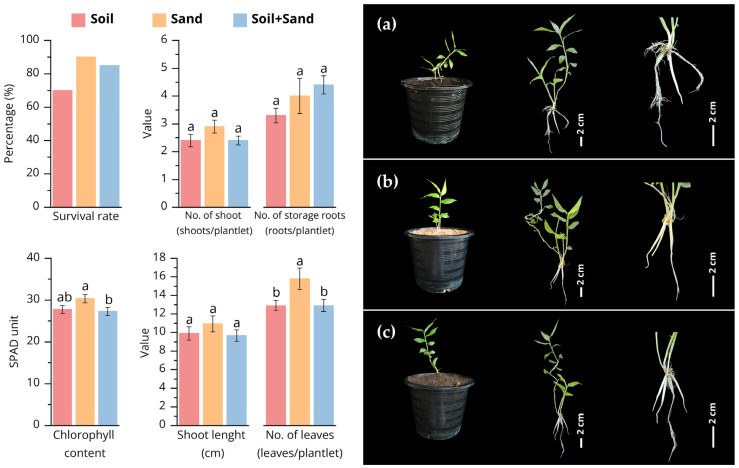
Comparative effects of planting substrates on the survival and growth performance of *Globba bicolor* plantlets after 8 weeks of cultivation. Bar graphs (**left**) illustrate mean values ± standard error (SE) based on ten replicates for growth parameters: survival rate (%), number of shoots (shoots/plantlet), number of leaves (leaves/plantlet), chlorophyll content (SPAD units), number of storage roots (roots/plantlet), and shoot length (cm). Different letters indicate statistically significant differences among treatments (DMRT, *p* < 0.05). Representative photographs (**right**) show morphological characteristics of plantlets grown in each substrate, including shoot development and root architecture: (**a**) soil; (**b**) sand; (**c**) soil-sand mixture. Scale bar = 2 cm.

This study evaluated the adaptation of *Globba bicolor* plantlets after in vitro cultivation. Healthy plantlets were transplanted onto different planting substrates for 8 weeks. Plantlets grown on sand exhibited the highest survival rate (90%) (Figure 4b), whereas those cultivated in soil (Figure 4a) and in a soil–sand mixture (Figure 4c) showed survival rates of 70% and 85%, respectively. In terms of shoot development, plantlets grown in sand produced the greatest number of shoots (2.90 ± 0.23 shoots/plantlet), but this was not significantly higher than those grown in soil (2.40 ± 0.22) and in the soil–sand mixture (2.40 ± 0.16) (Figure 4). Shoot elongation was enhanced in sand-grown plantlets, reaching an average length of 10.93 ± 0.85 cm. Leaf production was also greater in sand, with an average of 5.80 ± 0.35 leaves/plantlet (Figure 4). For root development, the number of storage roots was highest in the soil–sand mixture (4.40 ± 0.33 roots/plantlet), but differences across substrates were not significant. Root systems in all planting substrates appeared fibrous and well-distributed, as shown in the photographic comparisons (Figure 4a–c).

Physiologically, chlorophyll content measured by SPAD values was highest in sand-grown plantlets (30.34 ± 1.00 SPAD units), indicating better photosynthetic potential. Plantlets in soil and in the soil–sand mixture showed lower SPAD readings (27.75 ± 0.98 and 27.26 ± 0.98, respectively). Statistical analysis confirmed that sand substrate significantly improved growth parameters, as indicated by distinct letter groupings in the bar graphs (Figure 4). These results show that sand provides a more favorable environment for post-culture adaptation, supporting both shoot and root development as well as physiological vigor.

### 3.2. Phytochemical Profiling and Antioxidant Activity in Globba bicolor

#### 3.2.1. Total Phenolic Contents (TPC) and Total Flavonoid Contents (TFC)

**Figure 5:**
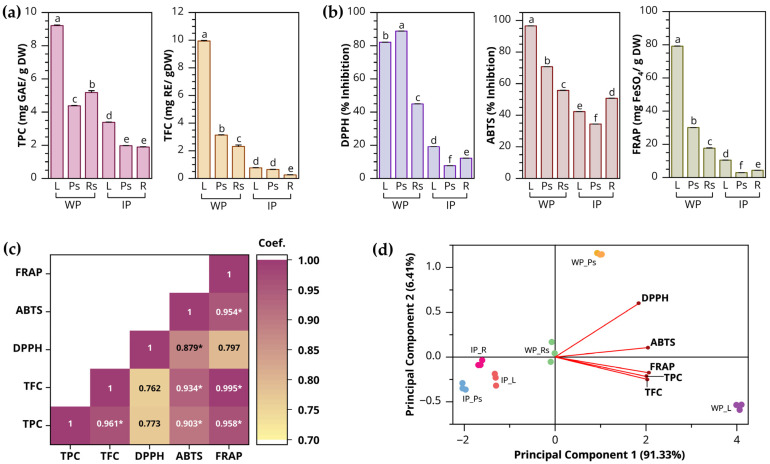
Evaluation of total phenolic content (TPC), total flavonoid content (TFC), and antioxidant activity of *Globba bicolor* ethanolic extract under different growth conditions. (**a**) TPC and TFC values; (**b**) antioxidant activity; (**c**) Pearson correlation coefficients (*r*); (**d**) biplot form of principal component analysis (PCA) scores and loading plot. TPC, TFC, and antioxidant activity (DPPH, ABTS, and FRAP) values represent the means of three replicates. Bars sharing the same letters are not significantly different at *p* ≤ 0.05 according to DMRT. Pearson correlation coefficients (*r*); * indicates statistical significance at *p* < 0.05 (two-tailed). Abbreviations: IP = in vitro-cultured plants; L = leaves; Ps = pseudostems; R = roots; Rs = rhizomes with storage roots; WP = wild plants.

The TPC and TFC of several plant parts under different growth conditions showed significant differences, as exhibited in Figure 5a. The TPC of both derived sources ranged from 1.89 to 9.21 mg GAE/g DW. The leaf extract from wild plants showed the highest value (9.21 mg GAE/g DW), followed by the rhizome with storage roots from wild plants (5.18 mg GAE/g DW). Furthermore, the leaves and pseudostems from in vitro-cultured plants exhibited 3.38 mg GAE/g DW and 1.97 mg GAE/g DW, respectively.

TFC values were markedly higher, with the greatest levels observed in the leaf segment. Across both sources, TFC ranged from 0.25 to 9.94 mg RE/g DW. In wild plants, the leaf extract displayed the maximum TFC value of 9.94 mg RE/g DW, whereas the pseudostems yielded 3.14 mg RE/g DW. In addition, the leaves and pseudostems of in vitro-cultured plants showed no significant differences, with values of 0.76 mg RE/g DW and 0.65 mg RE/g DW, respectively.

#### 3.2.2. Antioxidation Capacity

Antioxidation efficiency is defined as the capacity of bioactive compounds derived from biological sources to scavenge free radicals, suppress oxidative processes, and protect against diseases associated with oxidative stress [54]. The antioxidant activity of *Globba bicolor* grown under different conditions, including DPPH, ABTS and FRAP assay are displayed in Figure 5b. Notably, the ABTS assay revealed strong antioxidant capacity in wild plants, which was subsequently observed at lower levels in tissue-cultured plants. The inhibition values ranged from 34.42% to 96.51%. The highest ABTS radical scavenging activity was recorded in the leaf extract of wild plants (96.51% inhibition), followed by pseudostems (70.72% inhibition). In tissue-cultured plants, ABTS scavenging activity varied among organs, with inhibition values of 50.80% in roots, 42.29% in leaves, and 34.42% in pseudostems. Similarly, the DPPH assay showed radical scavenging activity ranging from 7.69% to 88.83% inhibition. Consistent with the ABTS results, wild plants exhibited higher antioxidant capacity than tissue-cultured plants. Among wild plant organs, pseudostems showed the highest DPPH inhibition (88.83%), followed by leaves (82.83%). In contrast, in vitro–cultured plants displayed considerably lower activity, with leaf segments exhibiting the highest scavenging activity (19.08%) compared to other organs. The FRAP assay further confirmed these findings by assessing the reducing power of the plant extracts through their ability to reduce Fe^3+^ to Fe^2+^. Higher FRAP values indicate stronger ferric reducing antioxidant capacity. Significant differences were observed among samples, with wild plants exhibiting markedly greater reducing efficiency than in vitro-cultured plants. Under wild growth conditions, the reducing power decreased in the order of leaves > pseudostems > underground organs, with leaves reaching 79.18 mg FeSO_4_/g DW. Conversely, in vitro-cultured plants showed a decreasing trend in the order of leaves > roots > pseudostems.

Pearson correlation analysis (Figure 5c) revealed strong positive relationships among phytochemical contents (TPC and TFC) and antioxidant activities (DPPH, ABTS, and FRAP), with correlation coefficients (*r*) ranging from 0.762 to 0.995. TPC was highly positively correlated with TFC (*r* = 0.961) and showed significant associations with ABTS (*r* = 0.903, *p* < 0.05) and FRAP (*r* = 0.958, *p* < 0.05), whereas its correlation with DPPH was moderate and not statistically significant (*r* = 0.773, *p* < 0.05). Similarly, TFC exhibited strong correlations with ABTS (*r* = 0.934, *p* < 0.05) and FRAP (*r* = 0.995, *p* < 0.05), but only a moderate association with DPPH (*r* = 0.762). Among antioxidant assays, ABTS was strongly correlated with FRAP (*r* = 0.954, *p* < 0.05) and DPPH (*r* = 0.879, *p* < 0.05), while the relationship between DPPH and FRAP was not statistically significant (*r* = 0.797, *p* < 0.05).

Principal component analysis (PCA) explained 97.74% of the total variance, with principal component 1 (PC1) accounting for 91.33% and principal component 2 (PC2) for 6.41% (Figure 5d). PC1 was predominantly influenced by TPC, TFC, ABTS, and FRAP, indicating that phenolic compounds were the primary contributors to antioxidant capacity. Wild leaf samples (purple dot) were positioned on the positive side of PC1, corresponding to their high phenolic and antioxidant values, whereas in vitro–cultured samples clustered on the negative side. This multivariate pattern supports the Pearson correlation results, confirming the close association among phenolic contents and antioxidant activities. The comparatively weaker contribution of DPPH to PC1 is consistent with its moderate correlation coefficients.

#### 3.2.3. FTIR-Based Spectral Characterization and Chemometric Prediction of Antioxidant Capacity

**Figure 6:**
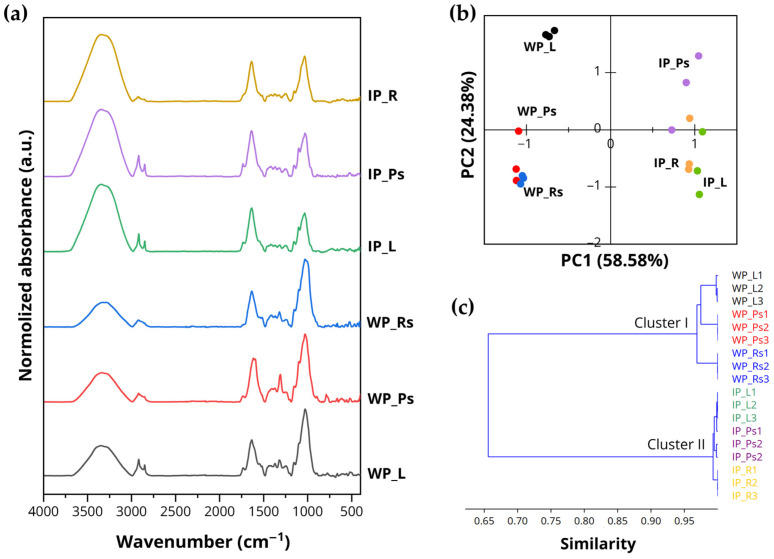
ATR-FTIR spectra analysis of *Globba bicolor* ground powder; (**a**) normalized average ATR-FTIR spectra ranging from 400 to 4000 cm^−1^; (**b**) principal component analysis (PCA) of ATR-FTIR spectra across different plant parts; (**c**) hierarchical cluster analysis (HCA) of ATR-FTIR spectra across different plant parts. Abbreviations: IP = in vitro-cultured plants; L = leaves; Ps = pseudostems; R = roots; Rs = rhizomes with storage roots; WP = wild plants.

The FTIR spectra revealed characteristic functional group vibrations of the plant sample powder, providing detailed information on the chemical bonding of bioactive compounds such as phenolics, flavonoids, cellulose, and other constituents. The representative ATR-FTIR spectra of various plant parts derived from different sources are shown in Table 3 and Figure 6a. A strong and broad absorption peak observed between 3336 and 3340 cm^−1^ is attributed to hydroxyl (O–H stretching) vibrations, characteristic of phenolics, flavonoids, and polysaccharides [55]. The absorption peak around 2852–2924 cm^−1^ can be attributed to the alkane group (C–H stretching vibration), indicating the presence of cellulose and hemicellulose components [56]. C=O stretching vibrations are observed at bands between 1732 and 1736 cm^−1^, corresponding to carbonyl groups belonging to esters and carboxylic acids present in lignin and phenolic acids [57]. The bands at 1616 cm^−1^ and 1636 cm^−1^ are attributed to aromatic group vibrations (C=C stretching), while those at 1516–1520 cm^−1^ correspond to C–C vibration (conjugated with C=C aromatic), indicating the presence of phenolic compounds [56]. Peaks around 1400–1460 cm^−1^ were assigned to –CH_2_ and –CH_3_ group vibrations (C–H bending), corresponding to phenolics and lignin components [58]. The vibration peak at 1368–1372 cm^−1^ could be ascribed to the bending of C–H and O–H in alcohol or phenols groups [57,59], while the peaks at 1248 cm^−1^ and 1252 cm^−1^ are attributed to C–O stretching vibrations of phenols groups and cellulose components [57,60]. A doublet peak was observed around 1110–1156 cm^−1^, corresponding to C–O stretching vibrations that could be attributed to aliphatic ethers in polysaccharide constituents [60]. Strong absorption peaks at 1028–1036 cm^−1^ correspond to C–O stretching vibrations of primary alcohol groups, which may be found in lignin [57].

PCA was applied to FTIR spectral metadata to reduce dimensionality while retaining the major variance. This facilitated clearer interpretation of structural patterns, variable interrelationships, and data distribution, as well as the identification of clustering trends among sample groups [61,62]. Methodologically, PCA projects correlated input variables into orthogonal dimensions, termed principal components (PCs). These components are sequentially extracted in descending order of explained variance, each constrained to be orthogonal to all previously derived components [63]. In this study, PCA was employed to classify plant parts originating from various sources using ATR-FTIR spectra, specifically within the spectral regions of 800–1800 cm^−1^ and 2800–3800 cm^−1^. The PCA score plot (Figure 6b) illustrates the clustering patterns among the samples. PC1, explaining 58.58% of the total variance, primarily accounted for the separation between wild and in vitro-cultured plant groups. PC2, which represented 24.38% of the variance, further discriminated among subgroups within both wild and in vitro-cultured samples. Together, PC1 and PC2 explained approximately 83% of the total variance, indicating that the major spectral variations were effectively captured by the model.

In agreement with the PCA results, the HCA dendrogram (Figure 6c) depicts the similarity relationships among samples based on their FTIR spectra. The samples were clearly separated into two main clusters corresponding to wild and in vitro-cultured plants. Within each cluster, further sub-clustering was observed according to plant organs, including leaves, pseudostems, and rhizomes/roots. Samples within the same category exhibited high similarity, whereas clear separation was evident among different sources and organs. The clustering patterns revealed that PCA and HCA clearly discriminated the samples based on their spectral characteristics.

**Figure 7:**
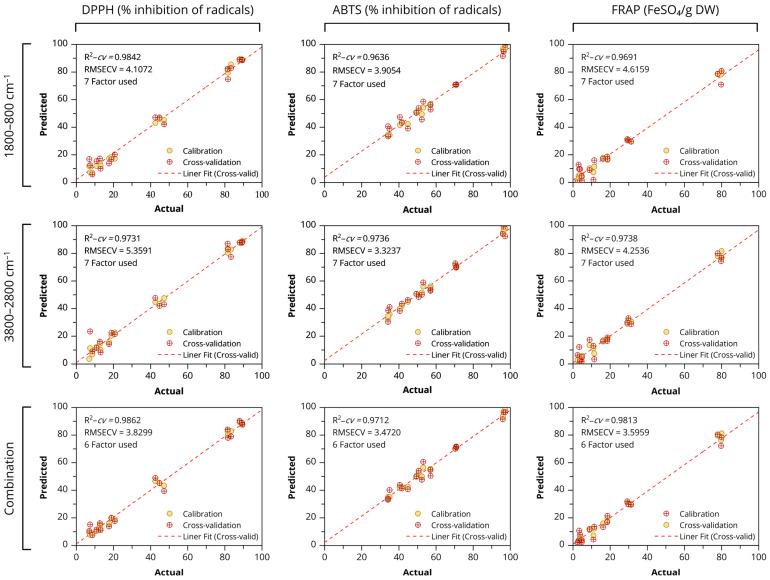
Scatter plots of ATR–FTIR–PLSR models using leave-group-out cross-validation (LGO) for predicting antioxidant activities (DPPH, ABTS, and FRAP assays) of *Globba bicolor* based on different spectral regions (1800–800 cm^−1^, 3800–2800 cm^−1^, and the combined range).

A multivariate approach was applied to predict the antioxidant activity of *Globba bicolor* from ATR-FTIR spectra using PLSR. The performance of the ATR–FTIR–PLSR models for DPPH, ABTS, and FRAP based on different spectral regions is summarized in Table 4 and Figure 7. All models showed strong calibration and validation performance, indicating that FTIR spectra contain relevant chemical information for estimating antioxidant capacity. For the fingerprint region (1800–800 cm^−1^), excellent calibration results were obtained, with R^2^–*cal* values between 0.9964 and 0.9970 and RMSEC ranging from 1.2347 to 1.8023. The corresponding cross-validation statistics remained high (R^2^–*cv* = 0.9636–0.9842), with RMSECV values of 3.9054–4.6159. Among the assays, the DPPH model exhibited the highest validation accuracy in this region (R^2^–*cv* = 0.9842 with 7 latent variables). Models developed using the functional group region (3800–2800 cm^−1^) also demonstrated strong performance (R^2^–*cal* = 0.9948–0.9968). Cross-validation with 7 latent variables yielded R^2^–*cv* values between 0.9731 and 0.9738, while RMSECV ranged from 3.3237 to 5.3591. The lowest RMSECV was obtained for ABTS (3.3237). Combining both spectral regions improved model robustness and reduced model complexity, as only 6 latent variables were required. The combined models produced high R^2^–*cal* (0.9939–0.9967) and enhanced validation statistics (R^2^–*cv* = 0.9712–0.9862; RMSECV = 3.4720–3.8299). In particular, the DPPH model achieved the best overall prediction (R^2^–*cv* = 0.9862, RMSECV = 3.8299), while FRAP also showed reliable performance (R^2^–*cv* = 0.9813, RMSECV = 3.5959). Models constructed using combined fingerprint and functional group spectral regions showed higher predictive performance than those using individual regions.

**Figure 8:**
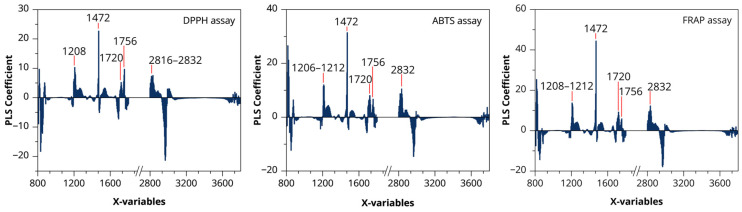
Regression coefficient plot of the PLSR model for antioxidant activity (DPPH, ABTS, and FRAP assays) based on the combined spectral ranges (1800–800 cm^−1^ and 3800–2800 cm^−1^) using six latent factors.

The PLS regression coefficient plots of the optimal ATR-FTIR–PLS models for antioxidant activity (DPPH, ABTS, and FRAP assays) are presented in Figure 8. The magnitude of each coefficient represents the relative contribution of individual wavenumbers to model predictions, with higher absolute values indicating stronger associations between predictors and responses. The sign of the coefficients denotes the direction of the relationship, where positive and negative values indicate positive and negative associations, respectively [64]. Across all three models, pronounced positive coefficients were consistently observed near 1472 cm^−1^, a region commonly assigned to C–H bending vibrations [58]. Additional influential coefficients were detected in the 1208–1212 cm^−1^ and 1260–1264 cm^−1^ regions, attributed to C–O stretching and C–O–C vibrations [57,60]. Positive coefficients were also present within the 1536–1756 cm^−1^ range, including bands around 1720 and 1756 cm^−1^, which are associated with conjugated C=O stretching and amide-related vibrations [57]. In the high-wavenumber region, coefficients around 2816–2832 cm^−1^ were observed and assigned to symmetric and asymmetric C–H stretching of aliphatic groups [56]. The regression coefficient profiles of the DPPH, ABTS, and FRAP models exhibited similar spectral patterns, although differences in coefficient magnitude were observed among the assays, indicating variation in the relative contribution of specific wavenumber regions to each model.

#### 3.2.4. Phenolic Acids and Flavonoid Compounds in *Globba bicolor* Analyzed by HPLC

The quantitative and qualitative profiles of different plant parts of *Globba bicolor* extracts under various growth conditions were systematically evaluated and compared using HPLC analysis to characterize their chemical constituents (Table 5). In wild plants, leaf extracts exhibited the highest levels of most detected compounds. Cinnamic acid was abundant in leaves (2096.09 µg/g DW), followed by ferulic acid (1598.07 µg/g DW), syringic acid (1449.61 µg/g DW), and gallic acid (387.35 µg/g DW). Phenolic acid levels in pseudostems ranged from 36.85 to 342.33 µg/g DW, with gallic acid identified as the predominant constituent. Moreover, the rhizome part exhibited cinnamic acid at the maximum concentration (758.67 µg/g DW). However, vanillic acid and *p*-coumaric acid were mainly detected in pseudostems and rhizomes, whereas caffeic acid was not detected in any tissues. Among wild plant organs, flavonoid compounds in pseudostems and leaves showed the highest rutin content (1273.31 and 1190.11 µg/g DW, respectively), while rhizomes accumulated higher contents of quercetin (503.23 µg/g DW) and kaempferol (343.70 µg/g DW). However, the total phenolic and flavonoid content of leaves (7817.80 µg/g DW) was markedly greater than that of pseudostems (2358.62 µg/g DW) and rhizomes (2521.79 µg/g DW).

In vitro-cultured plants grown on MS medium supplemented with 2 mg/L BA and 0.5 mg/L NAA also exhibited distinct accumulation patterns. Leaves contained relatively high amounts of gallic acid (363.26 µg/g DW), ferulic acid (283.86 µg/g DW), and syringic acid (609.82 µg/g DW) for phenolic acid compounds. Pseudostems accumulated higher gallic acid, cinnamic acid, ferulic acid (343.49, 208.32, and 205.25 µg/g DW, respectively) and moderate levels of *p*-coumaric acid (170.62 µg/g DW). In contrast, Roots showed elevated levels of syringic acid (731.22 µg/g DW) and cinnamic acid (635.64 µg/g DW). Among the flavonoids in in vitro–cultured samples, quercetin was present at relatively high levels in all organs, ranging from 225.50 to 295.77 µg/g DW, with the highest concentration observed in root extracts. Catechin was also absent across all samples, including those from wild plants. Compared with wild plants, in vitro cultures generally exhibited reduced concentrations of most phenolic acid compounds, particularly rutin, quercetin, ferulic acid, and cinnamic acid. Nevertheless, the total content in in vitro roots (2625.71 µg/g DW) was comparable to that of wild rhizomes (2521.79 µg/g DW), indicating that tissue culture conditions still support the biosynthesis of antioxidant-related metabolites. All results are presented in Table 5.

**Figure 9:**
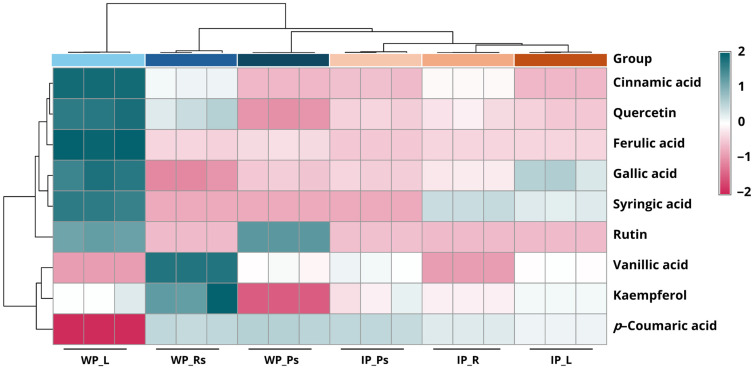
Hierarchical clustering heat map of the relative concentrations of phenolic acid and flavonoid compounds in *Globba bicolor* under different growth conditions, identified using HPLC. The data represents three replicates of the analysis. The color scale represents standardized relative concentrations (blue-green = high, red = low), and hierarchical clustering was performed using Ward’s linkage with Euclidean distance. Abbreviations: IP = in vitro-cultured plants; L = leaves; Ps = pseudostems; R = roots; Rs = rhizomes with storage roots; WP = wild plants. Caffeic acid and catechin were not included in this clustering analysis.

Figure 9 shows the hierarchical clustering heat map of relative concentrations of phenolic acids and flavonoid compounds in *Globba bicolor* determined by HPLC analysis. The heat map illustrates variation in the relative abundance of individual compounds across plant organs and growth sources, with standardized values represented by the color scale. Hierarchical clustering separated the samples into distinct clusters according to growth condition and organ type. Wild plant samples (WP) formed clusters that were clearly separated from in vitro–cultured plant samples (IP). Within the wild plant group, leaves (WP_L), rhizomes with storage roots (WP_Rs), and pseudostems (WP_Ps) samples exhibited distinct clustering patterns, indicating differences in phenolic and flavonoid profiles among organs. Similarly, in vitro–cultured samples clustered according to organ type, with leaves (IP_L), roots (IP_R), and pseudostems (IP_Ps) forming separate subclusters. The clustering of phenolic compounds also revealed compound-specific distribution patterns. Several phenolic acids and flavonoids displayed higher relative standardized values in wild plant organs, whereas lower or moderate relative values were observed in in vitro–cultured counterparts. Across all samples, variability in relative concentration patterns was evident among individual compounds, contributing to the observed sample separation in the hierarchical clustering.

#### 3.2.5. Volatile Oils Constituted of *Globba bicolor* Analyzed by GC-MS

**Figure 10:**
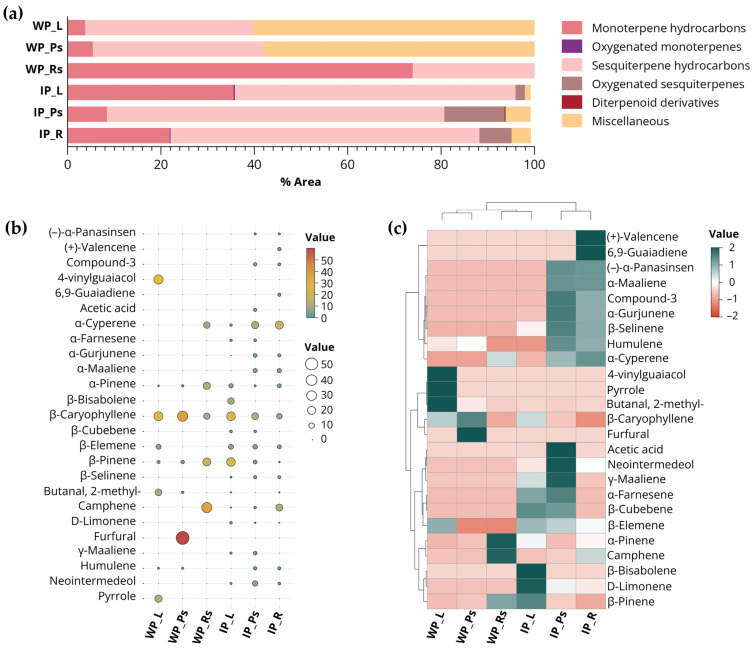
Profiling of volatile oils in different parts of *Globba bicolor* under various growth conditions, identified by GC–MS analysis; (**a**) profiling of the relative abundance of each terpene class (based on peak area); (**b**) balloon chart showing the detection of top 25 compounds in absolute %area values, based on the NIST 11 libraries (trace compounds < 0.5% were excluded from presentation); (**c**) heatmap clustering of the relative abundance of the top 25 compounds (%area ≥ 0.5). The color scale represents relative concentration. Hierarchical clustering was performed using Ward’s linkage with Euclidean distance. Abbreviations: IP = in vitro-cultured plants; L = leaves; Ps = pseudostems; R = roots; Rs = rhizomes with storage roots; WP = wild plants. Compound abbreviations; compound **3** = 2,6,10,10-tetramethylbicyclo (7.2.0) undeca-2,6-diene.

Volatile compounds of *Globba bicolor* were evaluated across different sources and plant parts using GC–MS analysis. A total of 90 volatile oil compounds were identified, including 12 compounds in wild plants and 87 compounds in in vitro cultured plants (details are provided in Table S1). In wild plant samples, particularly rhizomes with storage roots, a high proportion of terpenes was observed, consisting mainly of monoterpene hydrocarbons (73.94%), followed by sesquiterpene hydrocarbons (26.06%). In contrast, leaves and pseudostems predominantly accumulated sesquiterpene hydrocarbons, followed by monoterpene hydrocarbons (Table S1 and Figure 10a). In vitro propagated plants exhibited a broader diversity of terpene types, including monoterpene hydrocarbons, oxygenated monoterpenes, sesquiterpene hydrocarbons, oxygenated sesquiterpenes, and diterpene derivatives, indicating that in vitro culture conditions enhanced the production of diverse aromatic compounds. Across all plant parts grown under this condition, sesquiterpene hydrocarbons were the dominant constituents, accounting for 72.27% in pseudostems, 66.21% in roots, and 60.13% in leaves, followed by monoterpene hydrocarbons ranging from 21.87% to 35.56% (Table S1 and Figure 10a). Among the volatile compounds identified in in vitro cultured plants, diterpene derivatives were detected only in pseudostem segments at a low concentration (0.21%), whereas oxygenated monoterpenes were observed exclusively in leaf and root samples at low levels. Other constituents were classified as miscellaneous compounds and are listed in Table S1.

Figure 10b presents the 25 most abundant compounds identified in *G. bicolor* under both growth conditions. In wild plants, chemical profiles varied among plant organs. Leaves were dominated by 4-vinylguaiacol (28.16%), followed by β-caryophyllene (26.48%) and pyrrole (17.36%). In the pseudostems, furfural was the principal compound (56.20%), with β-caryophyllene (35.00%) and β-pinene (3.76%) present in lower proportions. In rhizomes and storage roots, volatile oils were prominent, particularly camphene (34.82%), β-pinene (20.90%), and α-pinene (17.00%). In contrast, in vitro cultured plants exhibited a more varied volatile composition. Leaves were mainly characterized by β-caryophyllene (25.21%), β-pinene (24.54%), and β-bisabolene (13.69%). The pseudostem segment was dominated by α-cyperene (17.28%), followed by β-caryophyllene (15.38%) and neointermedeol (9.48%). A similar pattern was observed in the root segment, where α-cyperene (21.53%) was also the most abundant compound, with camphene (15.54%) and β-caryophyllene (9.99%) occurring at lower levels.

The heatmap and hierarchical clustering analysis (Figure 10c) revealed clear differences in volatile compound profiles between wild and in vitro–cultured plants, with samples primarily grouped according to growth condition and, to a lesser extent, plant organ. Samples from wild plants, particularly leaves and pseudostems, tended to cluster together and were associated with higher relative abundances of specific compounds such as 4-vinylguaiacol, pyrrole, and several aldehyde-related volatiles. In contrast, underground samples of wild plants displayed a distinct chemical pattern characterized by stronger associations with monoterpene hydrocarbons, including α-pinene and camphene. In vitro–cultured samples formed a separate cluster, with pseudostem and root segments showing particularly similar chemical profiles. These tissues were more strongly associated with sesquiterpenes and oxygenated sesquiterpenes, including α-cyperene, neointermedeol, and related compounds, which occurred at comparatively higher levels than in wild plants. The results suggest that growth condition exerts a stronger influence than organ type on volatile composition, with in vitro cultivation associated with increased sesquiterpene-related compounds, while wild plants exhibit more organ-specific chemical signatures.

## 4. Discussion

### 4.1. Micropropagation of Globba bicolor on MS Medium Containing a Combination of PGRs

Plant growth regulators (PGRs) are well known to enhance plant growth and development in vitro by regulating shoot and root formation [65]. However, their effects depend on species and concentration. In this study, PGR treatments produced variable responses, highlighting the need for optimization. Shoot and root morphogenesis in *Globba bicolor* were influenced by the type and concentration of cytokinins (BA and kinetin) in combination with a low level of NAA. Among the treatments, BA combined with NAA was more effective than kinetin in promoting shoot multiplication and overall morphogenesis, while explants cultured on PGR-free medium showed limited response. Moderate BA concentrations (1.0–3.0 mg/L) combined with 0.5 mg/L NAA promoted vigorous shoot induction and root development, suggesting a synergistic interaction between cytokinin and auxin. These findings are consistent with tissue culture responses reported in other members of the Zingiberaceae [36,66,67], although optimal concentrations vary among species, reflecting genotype-specific hormonal sensitivity.

Cytokinins are known to stimulate cell division and shoot initiation, whereas low concentrations of auxin facilitate root initiation and help maintain the hormonal balance required for coordinated organ formation [65,68]. The superior performance of BA may be attributed to its higher stability in the culture medium and stronger ability to stimulate meristematic activity compared with kinetin. Previous studies have reported that BA exhibits greater cytokinin activity and persistence than kinetin, supporting its widespread use in ginger micropropagation systems [69,70]. The differential responses observed between BA and kinetin further indicate that cytokinin effects are not only concentration-dependent but also compound-specific. While BA primarily enhanced shoot proliferation, kinetin was more effective in promoting shoot elongation and balanced root development at low to moderate concentrations. This suggests that kinetin may preferentially stimulate cell elongation rather than intensive cell division, resulting in fewer but longer shoots and improved root growth, as reported in earlier cytokinin-specific studies [71].

At higher cytokinin concentrations (>3.0 mg/L), both BA and kinetin significantly reduced shoot and root formation. This inhibitory effect is likely associated with excessive hormonal levels leading to disrupted auxin transport and altered endogenous hormone homeostasis, thereby negatively affecting morphogenesis [72,73]. Excess cytokinin exposure has also been linked to abnormal tissue development and reduced differentiation potential in vitro, further supporting the dose-dependent nature of cytokinin action. Similar inhibitory responses have been reported in previous studies on *Globba* species [31,32,33,44]. This protocol supports balanced morphogenesis and may improve acclimatization success. Future studies should evaluate a wider range of PGRs, optimize lower hormone levels, and assess genetic stability to ensure long-term reliability. Although effective for the production and conservation of *G. bicolor*, the current system remains limited for large-scale industrial use. Further work should explore scalable systems, such as temporary immersion and bioreactor-based culture, to enable large-scale production.

### 4.2. Acclimatization and Substrate Adaptation of Globba bicolor Plantlets

The acclimatization results clearly demonstrate that substrate type plays a critical role in the successful transition of *Globba bicolor* plantlets from in vitro to ex vitro conditions. Among the tested substrates, sand provided the most favorable environment, resulting in the highest survival rate and superior vegetative growth. The enhanced performance of sand-grown plantlets can be attributed to their high porosity and good drainage, which likely improved root aeration and prevented excessive moisture accumulation, thereby reducing transplant stress during the early adaptation phase [74]. Although shoot number did not differ significantly among substrates, plantlets cultivated in sand exhibited enhanced shoot elongation and greater leaf production, indicating improved vegetative vigor.

Sand likely facilitated more efficient water and nutrient uptake, thereby supporting active shoot growth and leaf expansion during acclimatization. These findings are consistent with previous studies on *Globba sirirugsae*, which reported an 80% survival rate and successful plant development when sand was used as the planting substrate [36]. Furthermore, successful adaptation to sand substrate has been documented in several *Globba* species, with *G. schomburgkii* exhibiting an 80% survival rate and *G. globulifera* achieving 100% survival [32,34]. The higher SPAD values observed in sand-grown plantlets further support this interpretation, as increased chlorophyll content reflects improved photosynthetic capacity and physiological adjustment under ex vitro conditions [74,75].

Root development showed less pronounced differences among substrates, with fibrous and well-distributed root systems observed in all treatments. This uniform root morphology suggests that the in vitro-derived roots were functionally competent and capable of adapting to different substrate conditions. The slightly higher number of storage roots in the soil–sand mixture may reflect improved nutrient availability; however, this advantage did not translate into superior overall growth or survival compared with sand. Importantly, the successful acclimatization observed in this study can be directly linked to the quality of plantlets produced during the in vitro stage. Balanced shoot and root morphogenesis achieved under optimized cytokinin–auxin treatments likely contributed to the high survival rates and robust growth during ex vitro adaptation [35,66,73]. Together, the results demonstrate that sand supports effective acclimatization of *Globba bicolor* and validate the optimized in vitro protocol as a basis for successful establishment. Future studies should integrate acclimatization experiments with genetic stability assessments to ensure the reliability and consistency of plantlets under ex vitro conditions.

### 4.3. Evaluation of Total Phenolic Contents (TPC) and Total Flavonoid Contents (TFC) and Antioxidant Activity of Globba bicolor

To establish the broad potential of *Globba bicolor* for diverse applications, phytochemical evaluation was conducted to identify and characterize its bioactive compounds. This study demonstrates that phytochemical accumulation and antioxidant capacity in *Globba bicolor* are strongly influenced by growth conditions and plant organs. Wild plants consistently showed higher TPC, TFC, and antioxidant activities than in vitro-cultured plants, indicating that environmental factors such as light intensity, temperature fluctuations, and biotic interactions are key drivers of secondary metabolite biosynthesis [76]. Similar trends have been reported in other Zingiberaceae species [34,77]. Leaves showed the highest TPC and TFC across both plant sources, particularly in wild-grown plants, showing that photosynthetically active tissues are the primary sites of phenolic accumulation. Phenolics and flavonoids are closely associated with photoprotection and oxidative stress defense, which explains their preferential accumulation in leaf samples [78]. In contrast, the markedly lower TPC and TFC observed in in vitro-cultured plants may be attributed to the controlled growth environment, where limited exposure to external stimuli suppresses the activation of the phenylpropanoid pathway [79]. Nevertheless, certain abiotic stresses inherent to in vitro culture, such as nutrient imbalance, light density, and temperature, have been reported to act as elicitors that can stimulate secondary metabolite production under specific conditions [80].

The antioxidant assays were consistent with the observed phytochemical patterns, with wild plant extracts showing stronger radical scavenging and reducing capacities than in vitro-cultured samples. The stronger correlations of TPC and TFC with ABTS and FRAP, compared to DPPH, suggest that antioxidant activity in *Globba bicolor* is primarily governed by electron transfer-based mechanisms rather than hydrogen atom transfer reactions [81,82]. Similar assay-dependent responses have been widely reported in polyphenol-rich plant extracts [83]. Correlation analysis further revealed strong positive relationships between phenolic contents and antioxidant activities, particularly between TFC and FRAP, indicating that flavonoids play a major role in ferric reducing capacity. In addition, PCA clearly separated wild and in vitro-cultured samples, with PC1 mainly driven by TPC, TFC, ABTS, and FRAP, highlighting the key role of phenolic compounds in determining antioxidant potential. Similar correlation patterns and PCA trends have been widely reported in phytochemical studies of medicinal plants under different cultivation conditions [84,85].

These differences in total phenolic and total flavonoid contents are likely influenced by plant age, developmental stage, and growth environment, as well as other regulatory factors, rather than the in vitro system itself [86]. Therefore, comprehensive phytochemical profiling using advanced analytical techniques is required to identify individual compounds and to evaluate both the diversity and abundance of bioactive constituents, which are essential for assessing their potential applications in various fields. Moreover, additional strategies such as elicitation, light manipulation, or controlled stress induction may be required to enhance antioxidant compound induction in in vitro-derived *Globba bicolor*, as suggested in earlier studies on secondary metabolite optimization [87]. However, a limitation of this study is that chemical analyses were conducted using only three replicates, representing the minimum requirement for statistical analysis. Future studies should include a larger number of replicates to improve the robustness of the results.

### 4.4. Multivariable Analysis for Antioxidant Prediction Using an ATR-FTIR Spectra

FTIR, a rapid and non-destructive method, is widely applied in phytochemical studies to characterize functional groups and chemical fingerprints in plant materials. In phytochemical studies, FTIR spectra reflect the presence of phenolics, flavonoids, carbohydrates, and other bioactive compounds, allowing preliminary sample discrimination and qualitative assessment [37]. In this study, spectral differences among samples indicate variations in phytochemical profiles between plant sources and organs, suggesting that antioxidant capacity in *Globba bicolor* is closely linked to its chemical composition. Distinct grouping patterns observed among samples indicate that different plant parts and cultivation conditions accumulate different sets of bioactive compounds. These differences are likely associated with variations in phenolic and flavonoid content, which play a key role in antioxidant activity. The strong relationship between spectral features and antioxidant responses further supports the role of these compounds in determining the bioactive properties of the species.

Partial least squares regression (PLSR) is widely applied in chemometric studies, particularly for predicting antioxidant activity through calibration and validation modeling [88]. In this study, PLSR was used to establish predictive relationships between FTIR-derived spectral features and antioxidant responses. Model performance also depends on spectral region selection. The 3800–2800 cm^−1^ region showed lower accuracy, likely due to broad overtone bands and scattering effects. In contrast, the 1800–800 cm^−1^ fingerprint region yielded improved predictions because it contains fundamental vibrations associated with bioactive compounds, consistent with previous reports [38,39]. Moreover, combining spectral regions further enhanced model performance by incorporating complementary chemical information. Consistent with Irnawati et al., integrating FTIR spectral data (3050–2800 and 1790–650 cm^−1^) with PLSR calibration provided a reliable approach for correlating measured antioxidant values with predicted outcomes, demonstrating strong predictive performance (*R*^2^ = 0.9403) in pumpkin seed oils [39].

Among the antioxidant assays, DPPH showed the strongest association with spectral data, followed by ABTS and FRAP, reflecting differences in their underlying reaction mechanisms [38]. PLS regression coefficients further indicated that antioxidant prediction is primarily associated with phenolic-related functional groups, including C–H vibrations (~1472 cm^−1^), C–O/C–O–C stretching (1208–1264 cm^−1^), and conjugated C=O bands, all linked to phenolic structures governing antioxidant activity [38].

These findings highlight the utility of ATR-FTIR combined with PLS regression chemometrics as a rapid method for predicting antioxidant activity in *Globba bicolor*. This approach can serve as a valuable complementary tool for routine quality control and screening of plant materials, enabling efficient preliminary assessment of phytochemical quality across diverse applications, including pharmaceuticals, nutraceuticals, and food science. Nevertheless, the present study is limited by the relatively small number of datasets used for model training. Future studies should incorporate larger calibration datasets and independent external validation samples to further strengthen the reliability and applicability of the models.

### 4.5. High-Performance Liquid Chromatography (HPLC) for Quantitative Determination of Phenolic Acid and Flavonoid Constituents from Globba bicolor

To determine the contents of individual phenolic acids and flavonoid compounds, HPLC analysis with external standards was performed. The chromatographic profiles revealed clear organ-dependent variation in phenolic and flavonoid accumulation in *Globba bicolor*. In wild plants, leaves exhibited the highest total phenolic acid and flavonoid contents, consistent with reports indicating that photosynthetically active tissues often accumulate elevated phenylpropanoids due to light exposure and oxidative stress [76,79]. The increased levels of cinnamic, ferulic, and syringic acids in leaves further reflect active phenylpropanoid metabolism, as these compounds contribute to UV protection and antioxidant defense [89].

The enrichment of gallic acid in pseudostems and cinnamic acid in rhizomes and storage roots suggests differential allocation of phenolic metabolites among plant organs. Nevertheless, phenolic profiles are influenced by multiple factors, including species-specific metabolism, extraction solvent polarity, analytical methodology, and growth conditions. In particular, solvent selection can substantially affect both the qualitative and quantitative recovery of phenolics, even within the same tissue, and such effects may vary among species due to differences in chemical composition and metabolite solubility [77,90]. The high rutin levels in leaves and pseudostems, together with elevated quercetin and kaempferol in rhizomes and storage roots, support previous findings that flavonoid biosynthesis is spatially regulated, with metabolites differentially distributed according to tissue function [91].

In vitro–cultured plants generally exhibited lower concentrations of major phenolic acids and flavonoids than wild plants, likely reflecting reduced environmental stress under controlled conditions, which can limit phenylpropanoid accumulation [87]. However, the comparable total phenolic and flavonoid contents observed in in vitro roots suggest that plant growth regulators may sustain secondary metabolite biosynthesis in culture. Similar patterns have been reported in other medicinal plants, where in vitro–cultured organs retain the capacity to produce secondary metabolites, albeit at levels differing from wild-grown counterparts [36].

Collectively, these findings establish *Globba bicolor* as a rich and promising source of secondary metabolites with potential applications under both natural and in vitro cultivation conditions. Further studies evaluating broader bioactivities, including anti-inflammatory and antimicrobial effects, are warranted to fully assess its functional potential.

### 4.6. Volatile Oils Composition in Globba bicolor

Volatile oils are important secondary metabolites that contribute to plant defense, ecological interactions, and aromatic properties [92]. In *Globba bicolor*, the detection of aromatic compounds in different plant parts suggests that the composition of volatile constituents may vary according to organ type and growth condition, indicating potential differences in the regulation of volatile biosynthesis pathways. In wild plants, underground organs (rhizomes and storage roots) were predominantly characterized by monoterpene hydrocarbons, whereas aerial parts accumulated higher proportions of sesquiterpene hydrocarbons. In contrast, in vitro–cultured plants exhibited greater chemical diversity, including oxygenated monoterpenes, oxygenated sesquiterpenes, and minor diterpene derivatives. This shift, together with the predominance of sesquiterpene hydrocarbons and increased levels of compounds such as α-cyperene and neointermedeol, suggests that in vitro conditions may alter terpene biosynthesis pathways. These compositional differences indicate that growth conditions strongly influence terpene profiles, which may affect both aroma characteristics and the potential bioactivity of the plant.

The predominance of β-caryophyllene in several organs of *Globba bicolor* is particularly noteworthy, as this sesquiterpene is widely reported in aromatic and medicinal plants and is often associated with anti-inflammatory and antimicrobial properties [93]. Its consistent detection in both wild and in vitro-cultured samples suggests that it may represent a characteristic constituent of the species. Similar patterns of sesquiterpene dominance have been described in other members of the genus *Globba*, indicating possible chemotaxonomic relevance within the genus [36,94,95]. The occurrence of monoterpenes such as α-pinene, β-pinene, and camphene in underground organs is also significant, as these compounds contribute to fresh, resinous aroma, and pine-like notes are commonly associated with the sensory quality of essential oils [96]. Reports on related Zingiberaceae species have likewise described monoterpene-rich rhizome profiles [22,36,95], supporting the role of underground tissues as important reservoirs of volatile constituents in this genus. In addition, the detection of 4-vinylguaiacol and furfural in aerial parts from wild plants may further influence the overall aroma profile and contribute to distinctive sensory attributes.

These findings clarify the volatile composition of *Globba bicolor* and provide essential chemical information that may support its future use in food, pharmaceutical, and other natural product applications. Moreover, to better evaluate the biological potential of volatile oils, further studies on antimicrobial and antioxidant activities are required. In addition, different extraction methods should be explored to obtain and characterize active compounds, which may further support their use in various fields.

## 5. Conclusions

This study integrates in vitro propagation, phytochemical profiling, and antioxidant evaluation to provide a comprehensive assessment of *Globba bicolor*. An efficient micropropagation system was established using MS medium supplemented with PGRs. Sand substrate provided favorable conditions for plant growth during acclimatization and subsequent transplantation. Wild plants exhibited higher levels of secondary metabolites, while in vitro-derived plants showed distinct but lower phytochemical profiles. These results indicate that the current in vitro system is suitable for plant production and conservation, particularly for ornamental and sustainable use. However, for pharmaceutical-related applications requiring high levels of bioactive compounds, wild plants remain a more promising source. Further optimization of in vitro conditions is necessary to improve phytochemical accumulation, and elicitor-based or other stimulatory approaches should be explored in future studies. In addition, ATR-FTIR-based chemometric analysis offers a simple and rapid method for non-destructive screening of plant quality, making it suitable for routine evaluation. In conclusion, this study provides a practical protocol for rapid plant propagation and supports the sustainable utilization of *G. bicolor* as an ornamental plant and a potential source of natural bioactive compounds.

## Figures and Tables

**Table 1:** Effect of the combination of BA and NAA at different concentrations on plant regeneration of *Globba bicolor* after 8 weeks of culture.

Concentrations of PGR Combinations		No. Shoots (Shoots/Explant)	Shoot Length (cm)	No. Roots (Roots/Explant)	Root Length (cm)
BA (mg/L)	NAA (mg/L)				
0	0	3.43 ± 0.95 c	2.69 ± 0.25 c	3.85 ± 0.70 d	1.42 ± 0.15 c
0.5	0.5	7.70 ± 1.21 ab	5.53 ± 0.58 a	12.90 ± 2.46 abc	6.07 ± 0.66 ab
1.0	0.5	8.60 ± 1.08 ab	6.54 ± 0.74 a	17.70 ± 1.56 a	6.08 ± 0.88 ab
2.0	0.5	9.10 ± 1.30 a	5.00 ± 0.64 ab	12.40 ± 1.87 abc	7.69 ± 0.71 a
3.0	0.5	5.10 ± 0.90 bc	3.53 ± 0.61 bc	9.80 ± 1.24 bcd	4.27 ± 1.05 b
4.0	0.5	6.70 ± 1.17 abc	2.80 ± 1.44 c	13.60 ± 2.25 ab	3.46 ± 0.94 bc
5.0	0.5	6.80 ± 1.23 abc	3.23 ± 0.50 c	10.20 ± 2.07 bc	5.57 ± 1.03 ab
6.0	0.5	5.29 ± 1.36 bc	2.54 ± 0.81 c	7.00 ± 2.89 cd	3.36 ± 1.12 bc

Values represent the mean ± standard error of ten replicates per treatment. Different letters within a column indicate significant differences at *p* < 0.05 according to Duncan’s multiple range test (DMRT).

**Table 2:** Effect of the combination of kinetin and NAA at different concentrations on plant regeneration of *Globba bicolor* after 8 weeks of culture.

Concentrations of PGR Combinations		No. Shoots (Shoots/Explant)	Shoot Length (cm)	No. Roots (Roots/Explant)	Root Length (cm)
Kinetin (mg/L)	NAA (mg/L)				
0	0	3.50 ± 0.60 ab	2.65 ± 0.27 b	0.20 ± 0.89 c	0.38 ± 0.14 d
0.5	0.5	6.40 ± 1.23 a	2.19 ± 0.33 b	10.90 ± 2.50 a	1.65 ± 0.27 cd
1.0	0.5	4.70 ± 0.91 ab	5.02 ± 0.76 a	11.10 ± 2.80 a	6.12 ± 1.01 a
2.0	0.5	5.30 ± 1.14 ab	3.57 ± 0.63 b	9.20 ± 2.29 ab	4.07 ± 0.93 b
3.0	0.5	3.70 ± 0.73 ab	2.40 ± 0.27 b	4.50 ± 1.32 bc	1.26 ± 0.33 cd
4.0	0.5	2.90 ± 0.86 b	2.40 ± 0.29 b	4.10 ± 1.22 bc	1.85 ± 0.61 cd
5.0	0.5	4.90 ± 0.65 ab	3.46 ± 0.30 b	10.10 ± 1.86 ab	3.83 ± 0.92 b
6.0	0.5	4.90 ± 0.91 ab	3.29 ± 0.30 b	11.80 ± 2.37 a	2.74 ± 0.25 c

Values represent the mean ± standard error of ten replicates per treatment. Different letters within a column indicate significant differences at *p* < 0.05 according to Duncan’s multiple range test (DMRT).

**Table 3:** FTIR absorption peaks, functional group identification, and biochemical components of *Globba bicolor* powder.

Wavenumber (cm^–1^)						Functional Group Vibration	Representative Biochemical Component
Wild Plants			In Vitro-Cultured Plants (2 mg/L BA + 0.5 mg/L NAA)				
L	Ps	Rs	L	Ps	R		
3344	3336	3336	3340	3340	3340	O–H stretching	Alcohols, Phenols (Phenolics, Flavonoids, Polysaccharides)
2920	2920	2920	2920	2920	2924	C–H Stretching	–CH_2_ Methylene (Cellulose)
2852	2852	-	2852	2852	2852	C–H Stretching	–CH_2_ Methylene (Cellulose)
1732	1732	-	1732	1736	-	C=O Stretching	Ester, Aldehyde, Carboxylic acid (Lignin, Phenolic acids)
1636	1616	1636	1636	1636	1636	C=C Stretching	Aromatic (Phenolic compounds)
1520	-	1516	-	-	1516	C–C Stretching	Aromatic (Phenolic compounds)
-	-	-	1460	-	1452	C–H Bending	CH_2_, CH_3_ (Phenolics, Lignin)
1436	-	-	1444	-	-	C–H Bending	CH_2_, CH_3_ (Phenolics, Lignin)
1416	1404	1412	1420	1416	1416	C–H Bending	CH_2_, CH_3_ (Phenolics, Lignin)
1372	1372	1368	1372	1372	1372	C–H Bending, O–H Bending	–CH_3_, Alcohols, Phenols
1320	1312	1320	1316	1320	1332	O–H Bending, C–O Stretching	Phenolics, Flavonoids, Cellulose
1248	1248	1248	1248	1248	1252	C–O Stretching	Phenols, Cellulose
1156	1152	1152	1156	1156	1152	C–O Stretching	Aliphatic ether (Carbohydrate, Saccharide)
1100	1100	1100	1104	1100	-	C–O Stretching	Aliphatic ether (Carbohydrate, Saccharide)
1028	1032	1032	1036	1032	1036	C–O Stretching	Alcohol, Cellulose

Abbreviations: L = leaves; Ps = pseudostems; R = roots; Rs = rhizomes with storage roots.

**Table 4:** Statistical parameters of ATR–FTIR-PLSR models for antioxidant activity prediction across different spectral ranges of the *Globba bicolor* sample.

Spectral Range Model	Antioxidant Assay	LV *	Calibration		Cross-Validation (LGO)	
			*R*^2^–*cal*	RMSEC	*R*^2^–*cv*	RMSECV
1800–800 cm^−1^	DPPH	7	0.9970	1.8023	0.9842	4.1072
	ABTS	7	0.9964	1.2347	0.9636	3.9054
	FRAP	7	0.9969	1.4704	0.9691	4.6159
3800–2800 cm^−1^	DPPH	7	0.9965	1.9399	0.9731	5.3591
	ABTS	7	0.9968	1.1640	0.9736	3.3237
	FRAP	7	0.9948	1.8900	0.9738	4.2536
Combined	DPPH	6	0.9967	1.8710	0.9862	3.8299
	ABTS	6	0.9939	1.5981	0.9712	3.4720
	FRAP	6	0.9960	1.6607	0.9813	3.5959

*R*^2^–*cal* = coefficient of determination for calibration; *R*^2^–*cv* = coefficient of determination for cross-validation; RMSEC = root mean square error of calibration; RMSECV = root mean square error of cross-validation; LV = latent variable. * The optimum number of PLSR components (LV) was selected based on the minimum value of the root mean square error (RMSE) of validation to avoid over-fitting of the model.

**Table 5:** Contents of phenolic acids and flavonoids in different plant parts of *Globba bicolor* at various growth conditions determined by HPLC analysis.

Compound Names	Compound Concentration (µg/g of Dried Weight)					
	Wild Plants			In Vitro-Cultured Plants (MS + 2 mg/L BA + 0.5 mg/L NAA)		
	L	Ps	Rs	L	Ps	R
Gallic acid	387.35 ± 1.63 a	342.33 ± 0.57 d	330.73 ± 0.77 e	363.26 ± 2.06 b	343.49 ± 0.52 d	348.74 ± 0.32 c
Vanillic acid	ND	36.85 ± 1.53 c	110.58 ± 0.44 a	37.59 ± 0.58 c	41.57 ± 1.55 b	ND
Caffeic acid	ND	ND	ND	ND	ND	ND
*p*-Coumaric acid	ND	175.71 ± 1.10 a	170.26 ± 0.80 b	146.27 ± 0.18 d	170.62 ± 0.49 b	154.83 ± 0.68 c
Ferulic acid	1598.07 ± 3.94 a	309.84 ± 1.48 b	273.13 ± 6.83 cd	283.86 ± 1.13 c	205.25 ± 0.28 e	271.19 ± 3.29 d
Syringic acid	1449.61 ± 17.68 a	ND	ND	609.82 ± 8.77 c	ND	731.22 ± 13.24 b
Cinnamic acid	2096.09 ± 0.22 a	158.37 ± 6.39 e	758.67 ± 8.26 b	164.03 ± 1.89 e	208.32 ± 8.25 d	635.64 ± 0.11 c
Rutin	1190.11 ± 17.77 b	1273.31 ± 0.52 a	31.49 ± 0.16 d	36.93 ± 0.06 d	60.10 ± 0.31 c	33.69 ± 0.34 d
Kaempferol	183.28 ± 9.29 b	ND	343.70 ± 29.02 a	182.49 ± 0.12 b	161.20 ± 16.82 b	154.63 ± 0.96 b
Quercetin	913.29 ± 17.36 a	62.21 ± 0.25 e	503.23 ± 27.64 b	225.50 ± 11.24 d	242.18 ± 10.40 d	295.77 ± 16.69 c
Catechin	ND	ND	ND	ND	ND	ND
**Total content**	7817.80 ± 31.40	2358.62 ± 6.74	2521.79 ± 40.70	2049.75 ± 14.45	1432.73 ± 21.32	2625.71 ± 21.41

Values represent the mean ± standard error of three replicates per treatment. Different letters within a row indicate significant differences at *p* < 0.05 according to Duncan’s multiple range test (DMRT). ND = not detected. Abbreviations: L = leaves; Ps = pseudostems; R = roots; Rs = rhizomes with storage roots.

## Data Availability

All data supporting this study are included in the article and its Supplementary Information. Raw data are available from the corresponding author upon reasonable request.

## Supplementary Materials

The following supporting information can be downloaded at https://www.mdpi.com/article/10.3390/biology15100743/s1, Table S1: Volatile oil composition of *Globba bicolor*.
