# Supplementary material for: Micropropagation and Acclimatization of Globba bicolor Gagnep. with Phytochemical Profiling and Antioxidant Evaluation
Source: Biology (Basel). 2026 May 8;15(10):743. doi: 10.3390/biology15100743 (PMC13203872; doi:10.3390/biology15100743)
Supplement: Supplementary file 1 [file biology-15-00743-s001.zip › biology-4268162-supplementary.pdf]

biology

MDPI

Supplementary

Micropropagation and Acclimatization of *Globba bicolor* Gagnep. with Phytochemical Profiling and Antioxidant Evaluation

Table S1. Volatile oil composition of *Globba bicolor*

No.	RT	Compound	MF	MW	RI	PubChem CID	% Area					
							Wild plants			In vitro propagation plants		
							L	Ps	Rs	L	Ps	R
**Monoterpene hydrocarbons**												
1.	8.151	Tricyclene	$C_{10}H_{16}$	136.23	922	79035	-	-	1.22	0.20	0.07	0.47
2.	8.483	α-Pinene	$C_{10}H_{16}$	136.23	933	6654	0.88	1.64	17.00	5.98	1.27	4.76
3.	8.925	Camphene	$C_{10}H_{16}$	136.23	948	6616	-	-	34.82	0.41	1.97	15.54
4.	9.744	Sabinene	$C_{10}H_{16}$	136.23	974	18818	-	-	-	0.91	-	-
5.	9.829	β-Pinene	$C_{10}H_{16}$	136.23	977	440967	2.93	3.76	20.90	24.54	4.49	0.78
6.	10.313	β-Myrcene	$C_{10}H_{16}$	136.23	993	31253	-	-	-	0.65	0.05	-
7.	11.469	D-Limonene	$C_{10}H_{16}$	136.23	1030	440917	-	-	-	2.01	0.60	0.32
8.	12.109	(Z)-β-Ocimene	$C_{10}H_{16}$	136.23	1051	5320250	-	-	-	0.71	-	-
9.	12.405	γ-Terpinene	$C_{10}H_{16}$	136.23	1061	7461	-	-	-	0.07	-	-
10.	13.339	δ-2-Carene	$C_{10}H_{16}$	136.23	1092	79044	-	-	-	0.08	-	-
**Oxygenated monoterpenes**												
11.	13.762	Linalool	$C_{10}H_{18}O$	154.25	1105	6549	-	-	-	0.15	-	-
12.	19.608	Bornyl Acetate	$C_{12}H_{20}O_2$	196.29	1290	6448	-	-	-	-	-	0.17
13.	20.833	Myrtenyl acetate	$C_{12}H_{20}O_2$	194.27	1331	11435490	-	-	-	0.13	-	-
**Sesquiterpene hydrocarbons**												
14.	21.132	δ-Elemene	$C_{15}H_{24}$	204.35	1341	89316	-	-	-	0.08	0.05	-
15.	21.483	α-Cubebene	$C_{15}H_{25}$	204.35	1354	442359	-	-	-		0.08	-
16.	21.857	(3aS,4R,7R)-1,4,9,9-Tetra-methyl-5,6,7,8-tetrahydro-4H-3a,7-methanoazulene	$C_{15}H_{22}$	202.33	1367	74819450	-	-	-	-	-	0.12
17.	22.187	1,2,9,10-Tetradehydroaris-tolane	$C_{15}H_{22}$	202.33	1379	5321842	-	-	-	-	-	0.20
18.	22.240	α-Copaene	$C_{15}H_{24}$	204.35	1381	12303902	-	-	-	0.19	0.10	
19.	22.680	β-Elemene	$C_{15}H_{24}$	204.35	1389	6918391	8.13	-	-	7.80	6.61	4.93
20.	22.922	α-Cyperene	$C_{15}H_{24}$	204.35	1405	12308843	-	-	13.39	2.04	17.28	21.53
21.	23.102	γ-Caryophyllene	$C_{15}H_{24}$	204.35	1412	5281522	-	-	-	0.11	-	-
22.	23.300	β-Cadinene	$C_{15}H_{24}$	204.35	1420	10657	-	-	-	-	-	0.72
23.	23.305	(-)-Isoledene	$C_{15}H_{24}$	204.35	1420	15431199	-	-	-	0.09	0.67	-
24.	23.405	β-Caryophyllene	$C_{15}H_{24}$	204.35	1426	5281515	26.48	35.00	12.67	25.21	15.38	9.99
25.	23.693	γ-Muurolene	$C_{15}H_{24}$	204.35	1435	12313020	-	-	-	0.17	-	-
26.	23.791	γ-Elemene	$C_{15}H_{24}$	204.35	1439	12309452	-	-	-	0.11	-	-
27.	24.078	Selina-5,11-diene	$C_{15}H_{24}$	204.35	1450	91704179	-	-	-	0.08	0.44	1.00
28.	24.296	6,9-Guaiadiene	$C_{15}H_{24}$	204.35	1458	527113	-	-	-	-	-	3.19
29.	24.321	Humulene	$C_{15}H_{24}$	204.35	1460	5281520	1.24	1.60	-	-	3.66	3.09
30.	24.349	β-Bisabolene	$C_{15}H_{24}$	204.35	1460	10104370	-	-	-	13.69	-	-
31.	24.549	Alloaromadendrene	$C_{15}H_{24}$	204.35	1468	10899740	-	-	-	0.16	-	-
32.	24.758	(+)-Longifolene	$C_{15}H_{24}$	204.35	1476	1796220	-	-	-	-	0.14	0.41
33.	24.893	α-Gurjunene	$C_{15}H_{24}$	204.35	1481	15560276	-	-	-	0.08	4.47	3.23

 https://doi.org/10.3390/biology15100743

No.	RT	Compound	MF	MW	RI	PubChem CID	% Area					
							Wild plants			In vitro propagation plants		
							L	Ps	Rs	L	Ps	R
34.	24.968	(−)-α-Panasinsen	$C_{15}H_{24}$	204.35	1484	578929	-	-	-	-	1.51	1.45
35.	25.067	β-Cubebene	$C_{15}H_{24}$	204.35	1488	93081	-	-	-	2.22	2.04	-
36.	25.141	2-Isopropenyl-4a,8-dimethyl-1,2,3,4,4a,5,6,7-octahydronaphthalene	$C_{15}H_{24}$	204.35	1491	605019	-	-	-	0.85	-	1.09
37.	25.148	Aristolochene	$C_{15}H_{24}$	204.35	1491	161533	-	-	-	0.46	-	-
38.	25.217	β-Selinene	$C_{15}H_{24}$	204.35	1494	442393	-	-	-	0.95	3.34	2.62
39.	25.397	1H-Cyclopropa[a]naphthalene, decahydro-1,1,3a-trimethyl-7-methylene-, [1aS-(1aα,3aα,7aβ,7bα)]- or γ-Maaliene	$C_{15}H_{24}$	202.33	1501	595370	-	-	-	1.64	4.20	-
40.	25.400	(+)-Valencene	$C_{15}H_{24}$	202.33	1501	9855795	-	-	-	-	-	3.77
41.	25.542	α-Muurolene	$C_{15}H_{24}$	204.35	1507	12306047	-	-	-	0.17	-	-
42.	25.550	δ-Guaiene	$C_{15}H_{24}$	204.35	1507	94275	-	-	-	-	0.42	-
43.	25.700	α-Farnesene	$C_{15}H_{24}$	204.35	1513	5281516	-	-	-	1.93	2.37	-
44.	25.800	β-Chamigrene	$C_{15}H_{24}$	204.35	1517	442353	-	-	-	-	-	0.58
45.	25.898	γ-Cadinene	$C_{15}H_{24}$	204.35	1521	6432404	-	-	-	0.26	-	-
46.	26.009	α-Maaliene	$C_{15}H_{24}$	204.35	1526	12312615	-	-	-	-	5.79	5.66
47.	26.013	7-epi-α-Selinene	$C_{15}H_{24}$	204.35	1526	10123	-	-	-	1.05	-	-
48.	26.116	δ-Cadinene	$C_{15}H_{24}$	204.35	1530	12306054	-	-	-	0.79	-	-
49.	26.132	2,6,10,10- tetramethylbicyclo(7.2.0) undeca-2,6-diene	$C_{15}H_{24}$	204.35	1531	5367602	-	-	-	-	3.64	2.63
50.	28.632	Longipinane, (E)-	$C_{15}H_{26}$	204.35	1637	572863	-	-	-	-	0.08	-
**Oxygenated sesquiterpenes**												
51.	24.623	Ishwarol B	$C_{15}H_{24}O$	220.35	1471	91710043	-	-	-	-	0.44	1.52
52.	26.784	Elemol	$C_{15}H_{26}O$	222.37	1558	92138	-	-	-	-	0.17	-
53.	26.880	4,6,6-Trimethyl-2-(3-methylbuta-1,3-dienyl)-3-oxatricyclo[5.1.0.0(2,4)] octane	$C_{15}H_{22}O$	218.33	1562	571933	-	-	-	-	0.08	-
54.	27.461	Longipinocarvone	$C_{15}H_{22}O$	218.33	1586	535296	-	-	-	-	0.06	-
55.	27.639	Caryophyllene oxide	$C_{15}H_{24}O$	220.35	1593	1742210	-	-	-	0.30	0.79	-
56.	27.640	Ledol	$C_{15}H_{26}O$	222.37	1593	11074994	-	-	-	-	-	0.20
57.	27.865	epi-α-Muurolol	$C_{15}H_{26}O$	222.37	1603	3084331	-	-	-	-	0.18	-
58.	28.234	15-Hydroxy-α-muurolene	$C_{15}H_{24}O$	220.35	1619	14845386	-	-	-	0.05	0.12	0.50
59.	28.238	6-Isopropenyl-4,8a-dimethyl-1,2,3,5,6,7,8,8a-octahydro-naphthalen-2-ol	$C_{15}H_{24}O$	220.35	1619	594234	-	-	-	-	0.36	-
60.	28.344	Guaiyl acetate	$C_{17}H_{28}O_2$	264.40	1624	240122	-	-	-	-	0.29	-
61.	28.548	Cyperenone	$C_{15}H_{22}O$	218.33	1633	12308615	-	-	-	-	-	0.39
62.	28.558	1,2,3,4,5,6-Hexahydro-1,1,5,5-tetramethyl-2,4a-methanonaphtha-7(4aH)-one	$C_{15}H_{22}O$	218.33	1635	90971	-	-	-	-	0.14	-
63.	28.769	Isoshyobunone	$C_{15}H_{24}O$	220.35	1643	5318673	-	-	-	-	-	0.86
64.	28.938	2H-Cyclopropa[g]benzofuran, 4,5,5a,6,6a,6b-hexahydro-4,4,6b-trimethyl-2-(1-methylethenyl)-	$C_{15}H_{22}O$	218.33	1650	608879	-	-	-	-	-	0.61
65.	28.959	Eudesm-7(11)-en-4-ol	$C_{15}H_{26}O$	222.37	1651	6432454	-	-	-	0.15	-	-

**No.**	**RT**	**Compound**	**MF**	**MW**	**RI**	**PubChem CID**	**% Area**					
							**Wild plants**			**In vitro propagation plants**		
							**L**	**Ps**	**Rs**	**L**	**Ps**	**R**
66.	29.318	Neointermedeol	$C_{15}H_{26}O$	222.37	1668	11877394	-	-	-	1.33	9.48	2.36
67.	29.722	Longifolenaldehyde	$C_{15}H_{24}O$	220.35	1685	565584	-	-	-	-	0.22	0.20
68.	31.189	1,5-Epoxysalvial-4(14)-ene	$C_{15}H_{24}O$	220.35	1752	85669481	-	-	-	0.19	0.20	0.07
69.	31.314	2H-2,4a-Ethanonaphthalen-8(5H)-one, hexahydro-2,5,5-trimethyl-	$C_{15}H_{24}O$	220.35	1758	613138	-	-	-	-	0.37	-
70.	31.336	(7a-Isopropenyl-4,5-dimethyloctahydroinden-4-yl) methanol	$C_{15}H_{26}O$	222.37	1759	605599	-	-	-	-	-	0.17
**Miscellaneous**												
**Aliphatic aldehydes**												
71.	2.528	Butanal, 3-methyl-	$C_5H_{10}O$	86.13	678	11552	-	-	-	0.09	0.18	0.28
72.	2.590	Butanal, 2-methyl-	$C_5H_{10}O$	86.13	681	7284	14.82	1.80	-	0.45	-	0.47
73.	2.617	2,3-dimethylpentanal	$C_7H_{14}O$	114.14	683	61917	-	-	-	-	0.29	-
74.	3.047	Pentanal	$C_5H_{10}O$	86.13	706	8063	-	-	-	-	-	0.43
75.	3.048	Hexanal	$C_6H_{12}O$	100.16	807	6184	-	-	-	-	-	0.89
**Heterocyclic volatile compounds**												
76.	3.048	2-Ethylfuran	C_6H_8O	96.13	706	18554	-	-	-	0.17		-
77.	3.083	Acetic acid	$C_2H_4O_2$	60.05	708	176	-	-	-	-	3.30	-
78.	4.252	Pyrrole	C_4H_5N	67.09	765	8027	17.30	-	-	-	-	-
**Carboxylic acid**												
79.	6.475	Furfural	C5H4O2	96.08	843	7362	-	56.20	-	-	-	-
**Phenolic aromatic compound**												
80.	21.724	4-vinylguaiacol	C9H10O2	150.17	1362	332	28.16	-	-	-	-	-
**Norisoprenoid**												
81.	26.280	2(1H)-Naphthalenone, octahydro-4a,7,7-trimethyl-, cis-	C13H22O	194.31	1537	534662	-	-	-	-	-	0.84
82.	28.347	β-Ionone	C13H20O	192.3	1624	638014	-	-	-	0.08	-	-
83.	28.777	3-Butenal, 2-methyl-4-(2,6,6-trimethyl-2-cyclohexen-1-yl)-	C14H22O	206.32	1646	93952	-	-	-	0.07	0.40	-
**Homoterpene (Isoprenoid derivative)**												
84.	27.466	(3E,7E)-4,8,12-Trimethyltrideca-1,3,7,11-tetraene	C16H26	218.40	1586	6443227	-	-	-	0.11	-	-
**Homoterpenoids**												
85.	28.931	5H-3,5a-Epoxynaphth[2,1-c]oxepin, dodecahydro-3,8,8,11a-tetramethyl-	C18H30O2	278.40	1650	93639	-	-	-	-	0.59	-
86.	32.545	Ambrial	C16H26O	234.38	1816	14165630	-	-	-	0.12	0.49	1.16
**Aliphatic hydrocarbon**												
87.	30.142	n-Heptadecane	C17H36	240.5	1703	12398	-	-	-	0.09	-	-
**Non-terpene**												
88.	32.858	Isopropyl myristate	C17H34O2	270.50	1831	8042	-	-	-	-	0.06	-
**Diterpenoid derivatives**												
89.	33.276	6,10,14-Trimethyl-2-pentadecanone	C18H36O	268.50	1852	10408	-	-	-	-	0.12	-
90.	36.355	(E)-15,16-Dinorlabda-8(17),11-dien-13-one	C18H28O	260.40	2002	13994559	-	-	-	-	0.09	-

Chemical Class Distribution of Volatile Compounds	% Area					
	Wild plants			In vitro propagation plants		
	L	Ps	Rs	L	Ps	R
**Monoterpene hydrocarbons** [%]	3.81	5.40	73.94	35.56	8.45	21.87
**Oxygenated monoterpenes** [%]	-	-	-	0.28	-	0.17
**Sesquiterpene hydrocarbons** [%]	35.85	36.60	26.06	60.13	72.27	66.21
**Oxygenated sesquiterpenes** [%]	-	-	-	2.02	12.9	6.88
**Miscellaneous** [%]	60.34	58.00	-	1.18	5.31	4.07
**Diterpenoid derivatives** [%]	-	-	-	-	0.21	-
**Total identification**	100	100	100	99.17	99.14	99.20
**Number of constituents**	8	6	6	47	48	39

Abbreviations: IP = in vitro-cultured plants; L = leaves; MF = molecular formula; MW = molecular weight; Ps = pseudostems; R = roots; RT = retention time; Rs = rhizomes with storage roots; WP = wild plants. *Terpenes were categorized according to the compound class of terpenoids.
